# Lineage-specific regulation of imprinted X inactivation in extraembryonic endoderm stem cells

**DOI:** 10.1186/1756-8935-7-11

**Published:** 2014-06-20

**Authors:** Sarra Merzouk, Jane Lynda Deuve, Agnès Dubois, Pablo Navarro, Philip Avner, Céline Morey

**Affiliations:** 1 Mouse Molecular Genetics Laboratory, Pasteur Institute, 25 rue du Dr Roux, 75015 Paris, France; 2 Pasteur Cell, Pierre and Marie Curie University (UPMC), 25 rue du Dr Roux, 75015 Paris, France; 3 Present address: Pierre and Marie Curie University (UPMC), UMR7622, Institute of Biology of Paris-Seine (IBPS), 75005 Paris, France; 4 Present address: Epigenetics of Stem Cells Laboratory’, Pasteur Institute, 25 rue du Dr Roux, 75015 Paris, France; 5 Present address: Dynamics of Epigenetic Regulation, EMBL Monterotondo, Adriano Buzzati-Traverso Campus, Via Ramarini 32, 00015 Monterotondo, Italy

**Keywords:** Epigenetic, Single-cell analyses, Stem cells, X-inactivation

## Abstract

**Background:**

Silencing of the paternal X chromosome (Xp), a phenomenon known as imprinted X-chromosome inactivation (I-XCI), characterises, amongst mouse extraembryonic lineages, the primitive endoderm and the extraembryonic endoderm (XEN) stem cells derived from it.

**Results:**

Using a combination of chromatin immunoprecipitation characterisation of histone modifications and single-cell expression studies, we show that whilst the Xp in XEN cells, like the inactive X chromosome in other cell types, globally accumulates the repressive histone mark H3K27me3, a large number of Xp genes locally lack H3K27me3 and escape from I-XCI. In most cases this escape is specific to the XEN cell lineage. Importantly, the degree of escape and the genes concerned remain unchanged upon XEN conversion into visceral endoderm, suggesting stringent control of I-XCI in XEN derivatives. Surprisingly, chemical inhibition of EZH2, a member of the Polycomb repressive complex 2 (PRC2), and subsequent loss of H3K27me3 on the Xp, do not drastically perturb the pattern of silencing of Xp genes in XEN cells.

**Conclusions:**

The observations that we report here suggest that the maintenance of gene expression profiles of the inactive Xp in XEN cells involves a tissue-specific mechanism that acts partly independently of PRC2 catalytic activity.

## Background

During embryonic preimplantation development of female mice, starting at the four-cell stage, the paternal X chromosome (Xp) undergoes global epigenetic silencing associated with the establishment of imprinted X chromosome inactivation (I-XCI) [1-5]. In the inner cell mass (ICM) of the implanting blastocyst, the Xp is then reactivated and a random XCI of the Xp or the maternal X chromosome (Xm) occurs *de novo* during the formation of the epiblast that will subsequently give rise to the adult tissues [1,6]. In contrast, the extraembryonic lineages of the trophectoderm (TE) and the primitive endoderm (PrE) exhibit I-XCI of the Xp, which is maintained afterward in the derived lineages of the placenta and the yolk sac, respectively [7,8].

Many studies have concentrated on the characterisation of random XCI using the *ex vivo* model of ICM-derived female embryonic stem (ES) cells, the differentiation of which is accompanied by the onset of X inactivation. In these cells, XCI initiates through the *cis*-accumulation of the *Xist* noncoding RNA (ncRNA) on the future inactive X (Xi), followed by recruitment of Polycomb repressive complexes PRC2 and PRC1 and other epigenetic modifications, which, together, result in the progressive establishment of an inactive state characterised by its extreme stability (for review, see [9-11] and references therein).

In contrast, I-XCI in extraembryonic tissues has been less extensively analysed. Studies of developing embryos or trophoblast stem (TS) cells derived from the TE [12] have revealed that, similarly to the randomly inactivated X, the inactive Xp in the TE lineage is associated with *Xist* ncRNA coating, depletion of active epigenetic marks and enrichment for the repressive H4K20me1 mark, the PRC2-dependent H3K27me3 mark and hypermethylation of CpG islands [3,13-18]. Despite these cumulative regulatory locks ensuring the maintenance of Xp silencing, the inactive state in the TE seems to be less stable than that of adult somatic tissues because transient reactivation of some Xp-linked genes occurs spontaneously in both TS and TE cells [18]. As a corollary, a large number of X-linked genes are expressed from both X chromosomes in female TS cells [13]. Intriguingly, the magnitude and extent of this escape from I-XCI increase during TE differentiation into trophoblast giant cells, as revealed by an accrued frequency of reactivation of different Xp-linked transgenes and by reactivation of endogenous Xp loci [3,16,19-22]. This relaxed silencing is further exacerbated upon depletion of the PRC2 member EED, indicating that PRC2, possibly via its H3K27me3 catalytic activity, plays a role in the maintenance of I-XCI in the TE lineage [23,24]. Collectively, these results suggest that I-XCI is more plastic than random XCI and indicate the interest in an in-depth analysis of the stability of I-XCI in the PrE and its derivatives.

The PrE originates from the ICM and gives rise, after differentiation, to the visceral endoderm (VE) and parietal endoderm (PE) that line the yolk sac, two tissues which maintain an inactive Xp [8]. Extraembryonic endoderm (XEN) cells have been derived from the PrE and show many of the properties of PrE stem cells, including the ability to self-renew indefinitely *ex vivo* and to contribute in a lineage-appropriate manner *in vivo*, although undifferentiated XEN cells contribute predominantly to the PE and much less efficiently to the VE [25-27]. Whilst the inactive Xp in XEN cells is coated by the *Xist* ncRNA, it has been reported not to accumulate the PRC2 complex and associated H3K27me3 [26]. EED-mutant embryos, however, show increased and frequent reactivation of an Xp-linked green fluorescent protein (GFP) transgene in cells of both the VE and the PE [24]. X-linked GFP reexpression is also observed upon loss of *Xist* coating in the PE, suggesting that both *Xist* ncRNA and PRC2 activity are involved in the maintenance of Xp silencing in differentiated PrE cells [28].

In order to compare the characteristics of I-XCI in the PrE to the X-inactivation process occurring in other lineages, we combined two different approaches: (1) profiling of active and repressive chromatin features along the X chromosomes using both chromatin immunoprecipitation followed by chip hybridisation (ChIP-chip) and high-resolution immunofluorescence followed by fluorescent *in situ* hybridisation (immuno-FISH) studies and (2) an analysis of X-linked transcriptional activities at the level of single XEN cells by FISH on RNA (RNA-FISH) and reverse transcription followed by quantitative polymerase chain reaction (RT-qPCR). We observed that the Xp in XEN cells, in contrast to findings previously reported by other researchers, was globally enriched in H3K27me3 compared to the Xm. Intriguingly, we observed that the topological distribution of H3K27me3 on the inactive Xp territory did not coincide strictly with that of *Xist* ncRNA, suggesting that some Xp loci may harbour only low levels of H3K27me3. Indeed, similarly to what is observed in TS cells, a large number of X-linked genes (approximately 15%) lack H3K27me3 on both X chromosomes in XEN cells. These H3K27me3-depleted genes tend to be expressed from the inactive Xp in a significant number of cells, indicating that they escape from I-XCI. The frequency of escape, which appears to be extremely variable from gene to gene, was not significantly modified after depletion of H3K27 trimethylation using an EZH2-specific inhibitor. In addition, I-XCI profiles appear to be tightly maintained upon differentiation of XEN cells into VE cells. Our observations indicate that whilst widespread escape from I-XCI emerges as a common characteristic of extraembryonic stem cells, this escape from I-XCI is associated with a different subset of genes in each extraembryonic lineage.

## Results

### Inactive X chromosome territory is enriched in H3K27me3 in XEN cells

In this study, we analysed one male cell line (GHP7/7) and two independently derived female XEN cell lines (GHP7/9 and GHP7/3) [26]. All three cell lines harboured morphological features typical of XEN cells and expressed PrE-specific lineage markers (Additional file 1). The female cell lines carry an Xp of 129Sv.Pgk1a origin and an Xm of 129Sv origin, allowing the identification of allele origin through the extensive polymorphisms located in the large Pgk1a-derived region surrounding the *Xist* gene.

**Figure 1:**
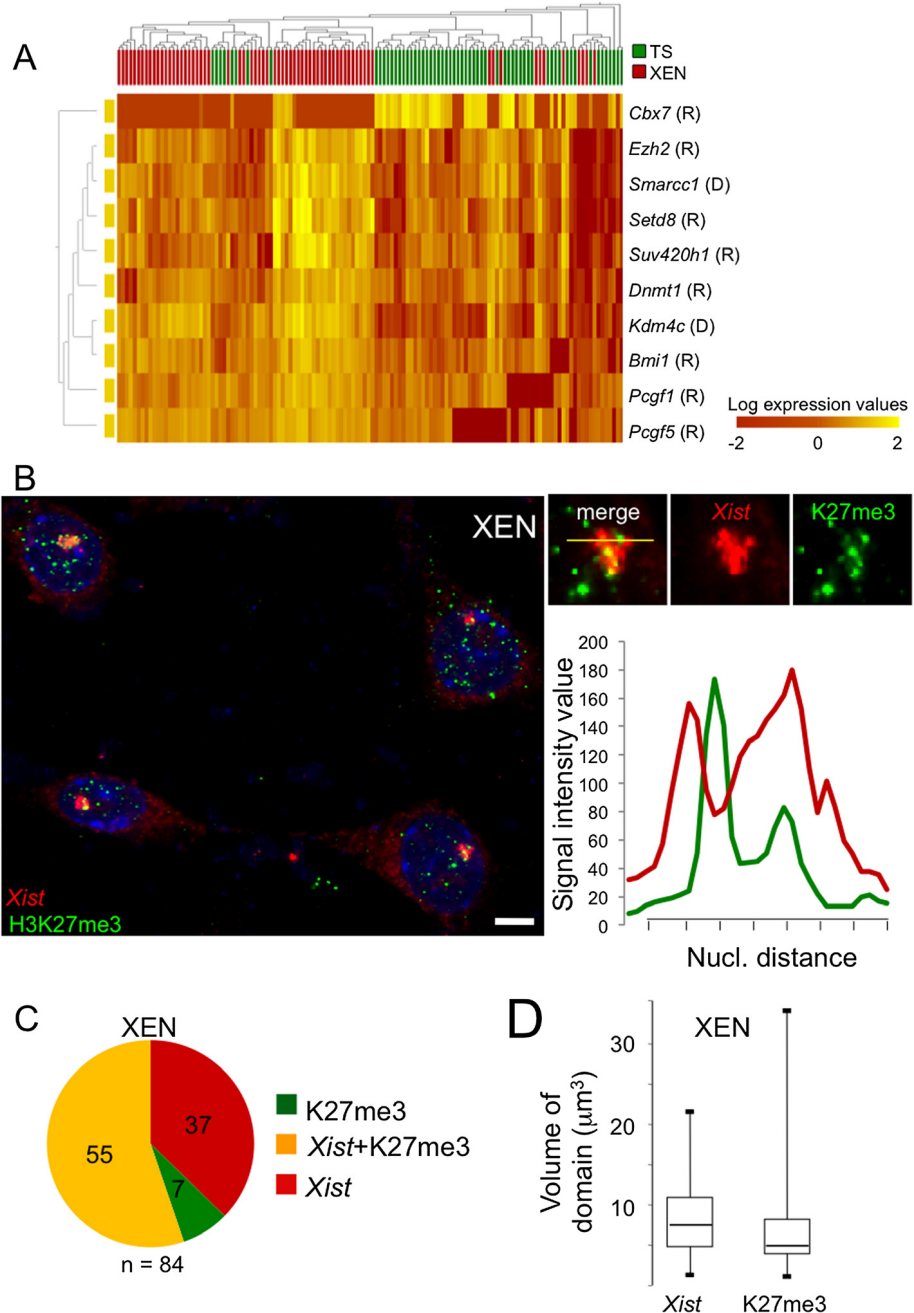
**H3K27me3 accumulates on the inactive X chromosome in female XEN cells. (A)** Heatmap of single-cell, steady-state RNA levels for the ten most stringent epigenetic discriminants for trophoblast stem (TS) cells and extraendoderm stem (XEN) cells (P < 5 × 10^−4^ by *F*-test). The complete data set is available in Additional file 2B. R, Regulator preferentially associated with transcription repression; D, Regulator associated with a dual function; it can be involved in transcription repression or activation. *n* = 72 female XEN cells (GHP7/9). **(B)** Representative image of immunofluorescence followed by fluorescence *in situ* hybridisation on RNA for H3K27me3 (green) and *Xist* (red) on female XEN cells (GHP7/3 cell line). Similar results were obtained with the GHP7/9 cell line (data not shown). Quantification of fluorescence intensities for *Xist* and H3K27me3 across the inactive X domain show that the two domains do not strictly overlap. Maximal projections after deconvolution are shown. Scale bar = 5 μm. **(C)** Pie charts showing the percentage of nuclei exhibiting an accumulation of *Xist* RNA only (red), coaccumulation of *Xist* RNA and H3K27me3 (yellow) and accumulation of H3K27me3 only (green) in female XEN cells (GHP7/3 cell line). **(D)** Boxplots showing the distribution of volumes occupied by *Xist* RNA and H3K27me3 on the inactive X in XEN cells (GHP7/3 cell line). The two distributions are significantly different. *P* < 0.05 by Kolmogorov–Smirnov test (*n* > 50). Vertical bars below and above the box-plots show the minimal and maximal values in the cell population respectively.

In a previous report, the authors indicated that I-XCI in XEN cells, unlike that in TS cells, is not accompanied by PRC2 recruitment and accumulation of associated H3K27me3 on the inactive Xp and suggested that XEN cells might express a distinct set of chromatin remodellers [26]. To address this question we compared, in XEN and in TS cells, the mRNA steady-state levels of 40 known epigenetic regulators using single-cell RT-qPCR analysis (Figure 1A and Additional file 2A and B). On the basis of their expression profiles, ten of the forty epigenetic regulators were able to discriminate between the two cell populations. These XEN/TS epigenetic discriminators included members of the PRC1 and PRC2 complexes. Unexpectedly, given the previously reported findings [26], mRNAs encoding the EZH2 H3K27 methyltransferase appeared more abundant, globally, in XEN cells than in TS cells.

This observation prompted us to reassess the distribution of H3K27me3 in XEN female cells using three-dimensional immuno-RNA-FISH for *Xist* and H3K27me3, followed by a deconvolution step (Figure 1B). *Xist* coating of the Xi was associated with a coenrichment in H3K27me3 in the majority of XEN cells, similarly to what we observed in TS cells (Figure 1C and Additional file 3). Although the volumes of H3K27me3 enrichment and of *Xist* domains on the Xi were not significantly different (Figure 1D), H3K27me3 and *Xist* nuclear foci did not show strict superimposability in the majority of XEN nuclei (Figure 1B), indicating that certain regions of the Xi, coated by *Xist*, may lack H3K27me3.

These results suggest that XEN and TS cells have distinct epigenetic identities. Notably, XEN cells highly express the *Ezh2* gene. Consistent with this, the Xi in XEN cells is globally enriched in H3K27me3.

### A large number of genes on paternal inactive X lack H3K27me3 in XEN cells

In order to confirm these results and identify the genomic regions of the inactive Xp showing lower levels of H3K27me3, we performed ChIP-chip analysis for this histone mark on male and female XEN cells using a high-resolution microarray covering the X chromosome and a subpart of chromosome 17, which served as a ChIP-chip efficiency control (see the Methods section for data treatment and quality controls). Comparing male and female profiles allowed us to detect differences marking specifically the Xi.

**Figure 2:**
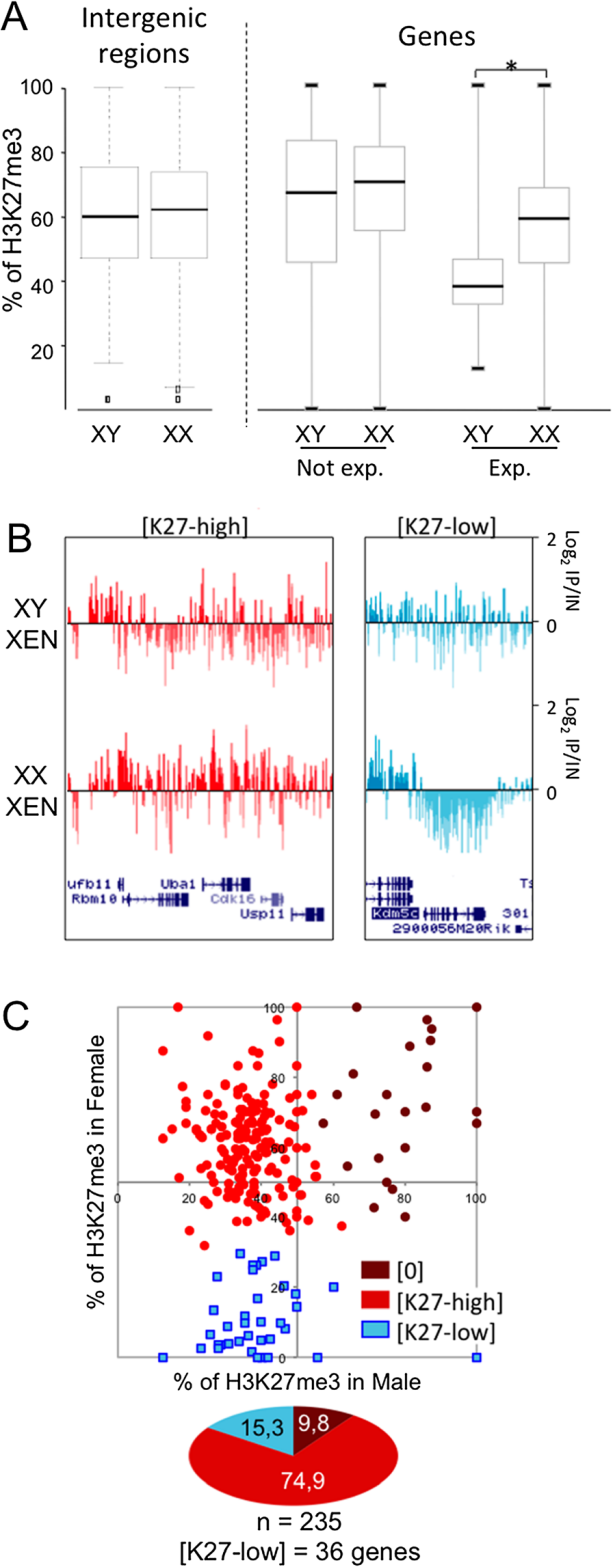
**Distribution of H3K27me3 on the X chromosome in XEN cells. (A)** Boxplots of the distribution of H3K27me3 along X-linked intergenic regions and X-linked gene bodies of expressed X-linked genes (Exp.) or along genes that are not significantly expressed (Not exp.) in male and female extraendoderm stem (XEN) cells (GHP7/7 and GHP7/9 cell lines). Expression data were extracted from the Gene Expression Omnibus database [GSE:15519] [29]. *n* = 1,054 intergenic regions and *n* = 642 X-linked genes. *P < 0.05 by Kolmogorov–Smirnov test. **(B)** Representative examples of H3K27me3 distribution along expressed genes enriched in H3K27me3 in female compared to male XEN cells ([K27-high]) and along expressed genes showing low levels of H3K27me3 in female and in male XEN cells ([K27-low]) (GHP7/7 and GHP7/9 cell lines). *mm9* University of California Santa Clara (UCSC) Genome Browser screenshots are shown. IP, immunoprecipitated DNA; IN, input DNA. **(C)** Scatterplot of H3K27me3 percentages along the body of expressed X-linked genes in female (*y*-axis) relative to male (*x*-axis) XEN cells (GHP7/7 and GHP7/9 cell lines (see Additional file 4A for results in the GHP7/3 cell line and Additional file 2C for the complete data set). Each dot represents a single gene and its respective percentage of H3K27me3 in male and female XEN cells. *k*-means clustering was applied, which led to the identification of three classes of genes. K27-high genes (red dots) are concentrated in the upper left quadrant consistently, with them being depleted in H3K27me3 in male cells and enriched in H3K27me3 in female cells. K27-low genes (blue dots) are significantly depleted in H3K27me3 in both male and female cells. In agreement with H3K27me3 marking preferentially the silent state, very few expressed genes are enriched in H3K27me3 in both male and female cells ([0] genes; indicated by maroon dots). These [0] genes may have resulted from the fact that gene expression data [29] and our chromatin immunoprecipitation followed by chip hybridisation experiment data were obtained from different XEN cell lines. The pie chart underneath the scatterplot represents the percentage of expressed genes in each H3K27me3 class.

X-linked genes, but not intergenic intervals, appeared significantly more enriched in H3K27me3 in female compared to male XEN cells, indicating that genes constitute the preferential targets of H3K27me3 accumulation on the Xi (Figure 2A). In addition, the majority of expressed genes, as opposed to silent genes, exhibited higher levels of H3K27me3 in female compared to male XEN cells. This is suggestive of an accumulation of this modification on Xi alleles, although we noticed that some expressed genes lacked H3K27me3 in both male and female extraembryonic cells, which is indicative of biallelic H3K27me3 depletion (Figure 2B). We were able to identify these H3K27me3-depleted Xi genes (H3K27me3-low) using unbiased *k*-means clustering based on the statistical comparison of H3K27me3 percentages along gene bodies in male and female XEN cells (see Figure 2C, the Methods section and Additional files 2C and 4A). Approximately 15% of expressed genes exhibited such a profile in a female XEN cell line (GHP7/9), as opposed to 19.5% that showed similar behaviour in TS cells and only about 7% in liver cells (Figure 2B and Additional file 4B and C). Amongst these H3K27me3-low genes, 19 were common to both XEN and TS female cells, and of those, only 2 were associated with reduced levels of H3K27me3 in the female liver (Additional file 4C).

We then assessed whether this lack of H3K27me3 on specific Xi genes in female XEN cells was associated with a gain of active histone mark H3K4me2 using a similar ChIP-chip approach. Lowered H3K4me2 levels in female XEN cells than in male XEN cells were observed at the majority of expressed genes, consistent with an absence of active histone marks on Xi alleles (Additional file 5A). In contrast, H3K27me3-low Xi genes appeared significantly enriched in H3K4me2 compared to H3K27me3-high Xi genes (*P* = 0.037 by Kolmogorov–Smirnov test) (Additional file 5B and C). We noted, however, that this effect was not observed at all H3K27me3-low genes and that some H3K27me3-high genes may also show high levels of H3K4me2 in female cells, indicating that the anticorrelation between H3K4me2 and H3K27me3 is not strict.

On the basis of these ChIP-chip analyses, we conclude that both the active and repressive histone marks H3K4me2 and H3K27me3 are distributed in a lineage-specific manner on Xi genes. Many of the genes lacking H3K27me3 on the Xi of XEN cells specifically showed a gain of H3K4me2 compared to Xi genes enriched in H3K27me3, indicating that some of these H3K27me3-low genes adopted the characteristics of active chromatin.

### Genes lacking H3K27me3 in female XEN cells escape from imprinted XCI to various degrees

**Figure 3:**
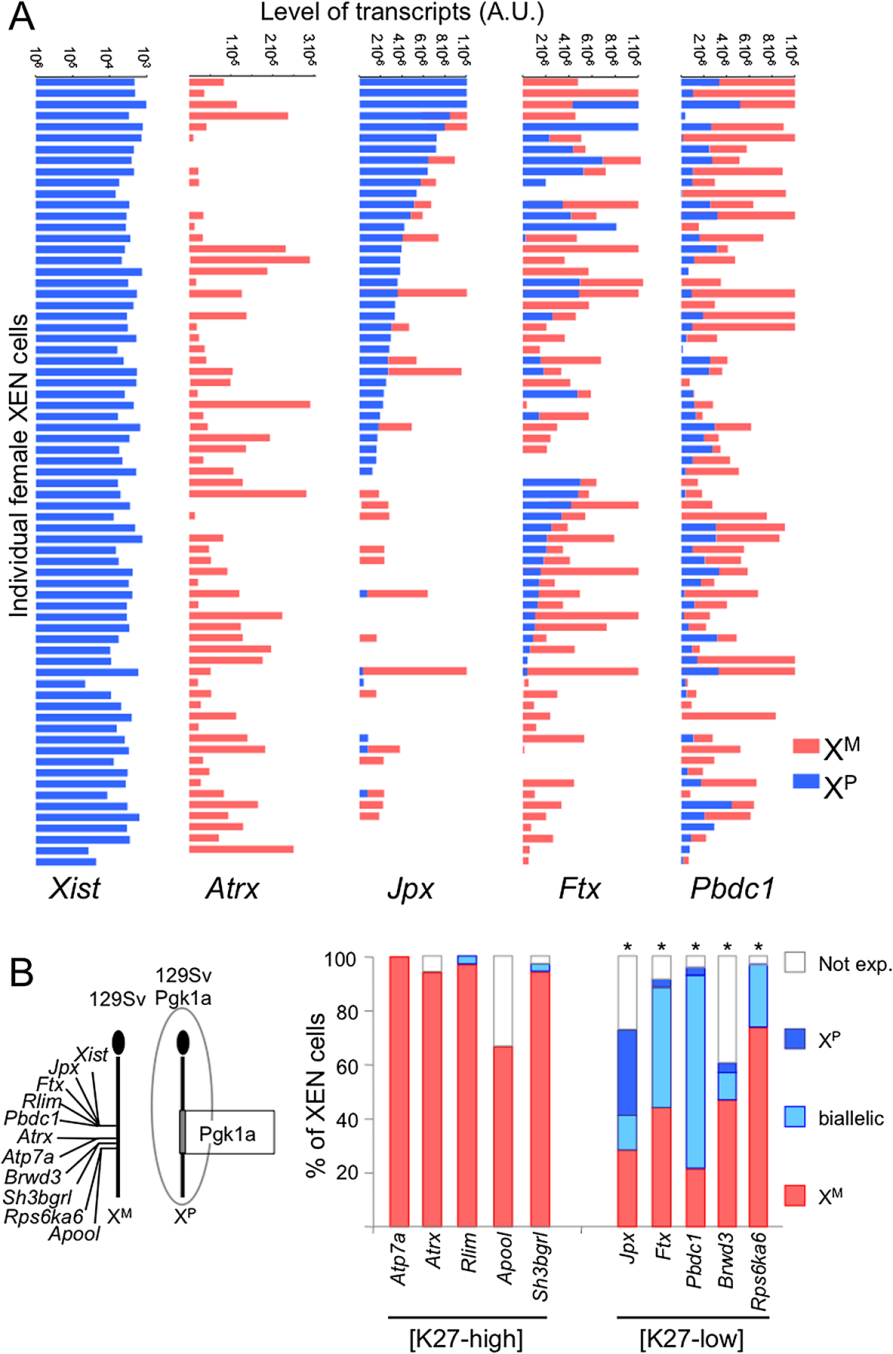
**RT-qPCR analysis of X-linked gene expression in single female XEN cells. (A)** Cumulative histograms showing allelic gene expression levels of *Xist*, *Atrx*, *Jpx, Ftx* and *Pbdc1* in the 72 individual extraendoderm stem (XEN) cells (GHP7/9 cell line). AU, Arbitrary unit. Logarithmic scale is used for *Xist*. **(B)** Cumulative histograms of the percentages of female XEN cells showing expression from the inactive paternal X (Xp) (dark blue), from the active maternal X (Xm) (red) or from both X chromosomes (light blue) for each indicated gene. X-linked genes are grouped according to their H3K27me3 enrichment in female XEN cells. The asterisks mark significant differences found upon analysis of the expression profile of a given gene showing low levels of H3K27me3 [K27-low] compared to any of the genes enriched in H3K27me3 [K27-high] analysed (*P* < 0.05 by Χ^2^ test). On the left, the diagram depicts the position of analysed genes along the X chromosome. The Pgk1a polymorphic region is shown in grey.

In order to address whether these active chromatin features are associated with gene expression from the Xi in XEN cells, we performed two types of analyses. First, in single XEN cells, we measured by allele-specific RT-qPCR, the maternal and paternal expression of five genes with high H3K27me3 levels (*Atp7a*, *Atrx*, *Rlim*, *Apool* and *Sh3bgrl*) and five genes with low H3K27me3 levels (*Ftx*, *Jpx*, *Pbdc1* (also known as *2610029G23Rik*), *Brwd3* and *Rps6ka6*), as well as *Xist* transcripts (see Additional file 6A for RT-qPCR controls). As expected in cells subjected to I-XCI, *Xist* expression appeared to be restricted to the Xp and genes showing high H3K27me3 levels were expressed predominantly from the active Xm (Figure 3). All five genes lacking global H3K27me3, on the other hand, showed a significant level of paternal expression in XEN individual cells, indicating that these genes escaped from I-XCI (Figure 3). We noted that the relative levels of expression from both the Xp and the Xm were extremely variable, both amongst cells and between escaper loci, leading to a highly heterogeneous XEN cell population. Consistent results were obtained with mixed cell populations (Additional file 6B).

**Figure 4:**
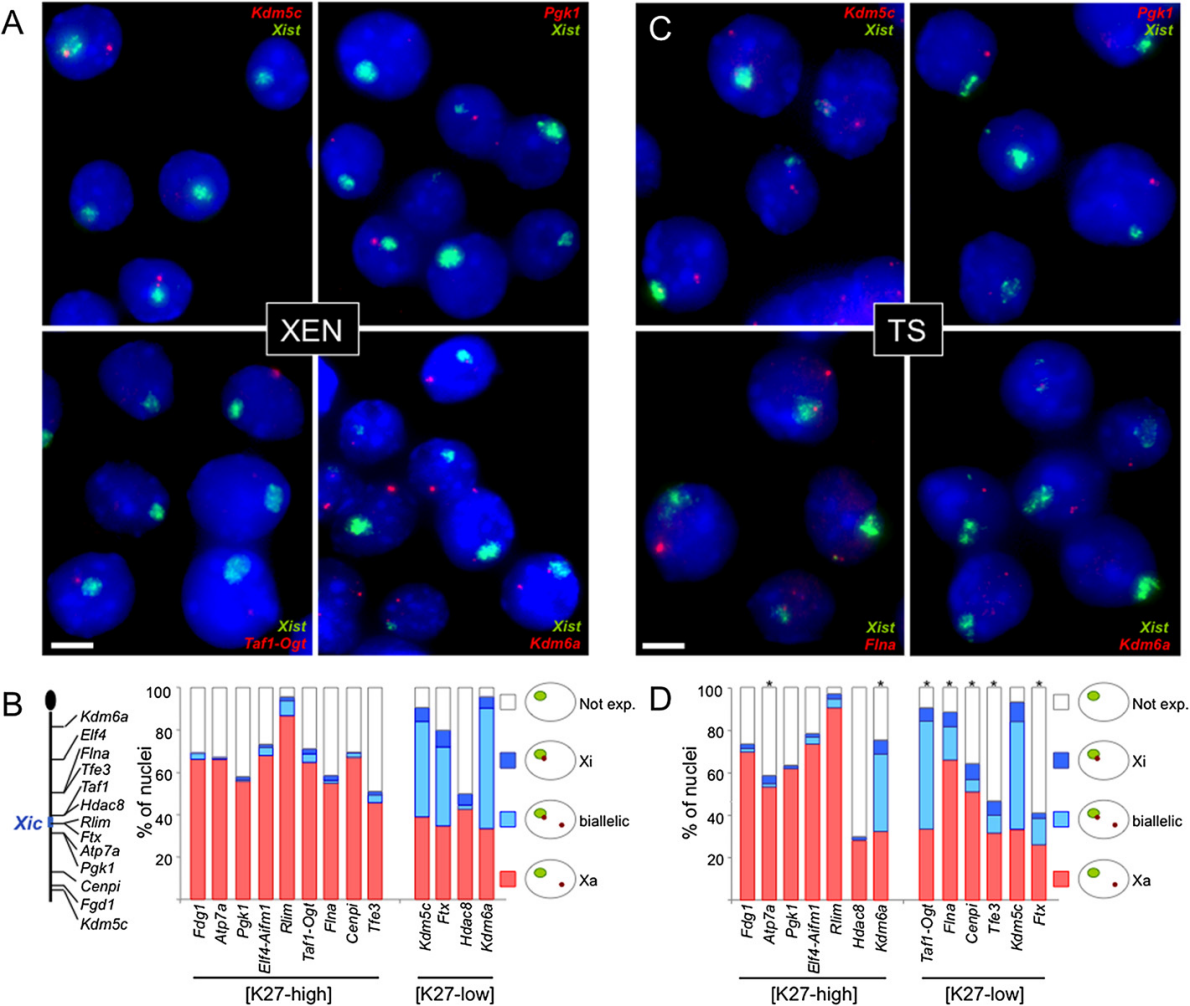
**Fluorescence *in situ *hybridisation on RNA analysis of X-linked gene expression in XEN compared to TS female cells. (A)** Representative images of fluorescence *in situ* hybridisation on RNA (RNA-FISH) analysis in extraendoderm stem (XEN) female cells (GHP7/9 cell line). The inactive X chromosome is identified through *Xist* RNA accumulation (green). Primary transcription at the indicated X-linked gene is codetected in red. Scale bar = 5 μm. **(B)** Cumulative histograms of the percentages of nuclei with the depicted expression pattern are shown. X-linked genes are grouped according to their H3K27me3 class. [K27-high], Enriched in H3K27me3 in female compared to male XEN cells; [K27-low], Low levels of H3K27me3 in female and in male XEN cells. On the left of the histogram, the diagram shows the localisation of RNA-FISH probes along the X chromosome. *n* > 200. **(C)** The same types of data shown in **(A)** are given for female trophoblast stem (TS) cells (F3 cell line). **(D)** The same types of data shown in **(B)** are given for female TS cells. *P < 0.05 by Χ^2^ test for significant differences with expression profiles in XEN cells (*n* > 200).

Second, we performed RNA-FISH on 13 different X-linked regions: 9 regions showing high levels of H3K27me3 (*Fgd1*, *Atp7a*, *Pgk1*, *Elf4-Aifm*, *Taf1-Ogt*, *Flna*, *Cenpi*, *Tcfe3* and *Rlim*) and 4 genes showing low levels of H3K27me3 (*Kdm5c*, *Ftx*, *Hdac8* and *Kdm6a*) (Additional file 2D). Importantly, some of these regions (namely, *Taf1-Ogt*, *Flna*, *Cenpi*, *Tcfe3, Hdac8 and Kdm6a*) displayed different H3K27me3 and H3K4me2 levels in XEN than in TS cells (Additional file 2C). The comparison of RNA-FISH expression profiles between these two extraembryonic cell types allowed us to assess the lineage specificity of Xp gene silencing in extraembryonic lineages (Figure 4).

In agreement with our chromatin profile analysis, most H3K27me3-high Xi genes in each extraembryonic cell type were transcribed predominantly from the Xa (*Xist*-lacking X) and not from the Xi (*Xist*-coated X), indicating that these genes are subject to I-XCI. H3K27me3-low genes, on the other hand, appeared to be transcribed from the Xi in a significant percentage of nuclei in both cell types, indicating that they escaped from I-XCI (Figure 4). Two exceptions were observed. *Hdac8*, which we expected to escape from I-XCI in XEN cells based on its H3K27me3 profile, showed no significant expression from the Xi in these cells (Figure 4B). This may be related to its globally highly reduced expression level (low steady-state level of mature transcripts [29]), which may introduce some stochasticity into transcription initiation. Conversely, the *Kdm6a* gene is transcribed from the Xi in a significant percentage of TS nuclei, despite showing significant levels (about 47%) of H3K27me3 on the Xi in this cell line (Figure 4D). However, the proportion of nuclei showing this behaviour was significantly smaller than that seen in female XEN cells in which *Kdm6a* was poorly enriched (about 10%) in H3K27me3. *Kdm6a* is known to robustly escape from XCI in somatic tissues [30].

As previously described for TS cells [13] and other adult cell types [31], we observed that maternal allelic expression prevailed over that of the paternal allele in XEN cells for all the escapers examined, suggesting that, for most XCI escapers, the escape mechanism was not fully efficient in relieving the inactivation. Indeed, the degree of escape, as estimated both by the number of nuclei exhibiting transcriptional activity from the Xi detectable by RNA-FISH and by the number of cells showing significant paternal expression detectable by single-cell RT-qPCR, appeared to be extremely variable, not only between cell types (for example, *Ftx* showed different levels of escape in TS cells compared to XEN cells) but also from gene to gene. This supports the idea that expression from the Xi at loci escaping from I-XCI is a locally regulated phenomenon.

### Level and extent of escape from imprinted XCI are not drastically modified upon inhibition of H3K27me3 in XEN cells

**Figure 5:**
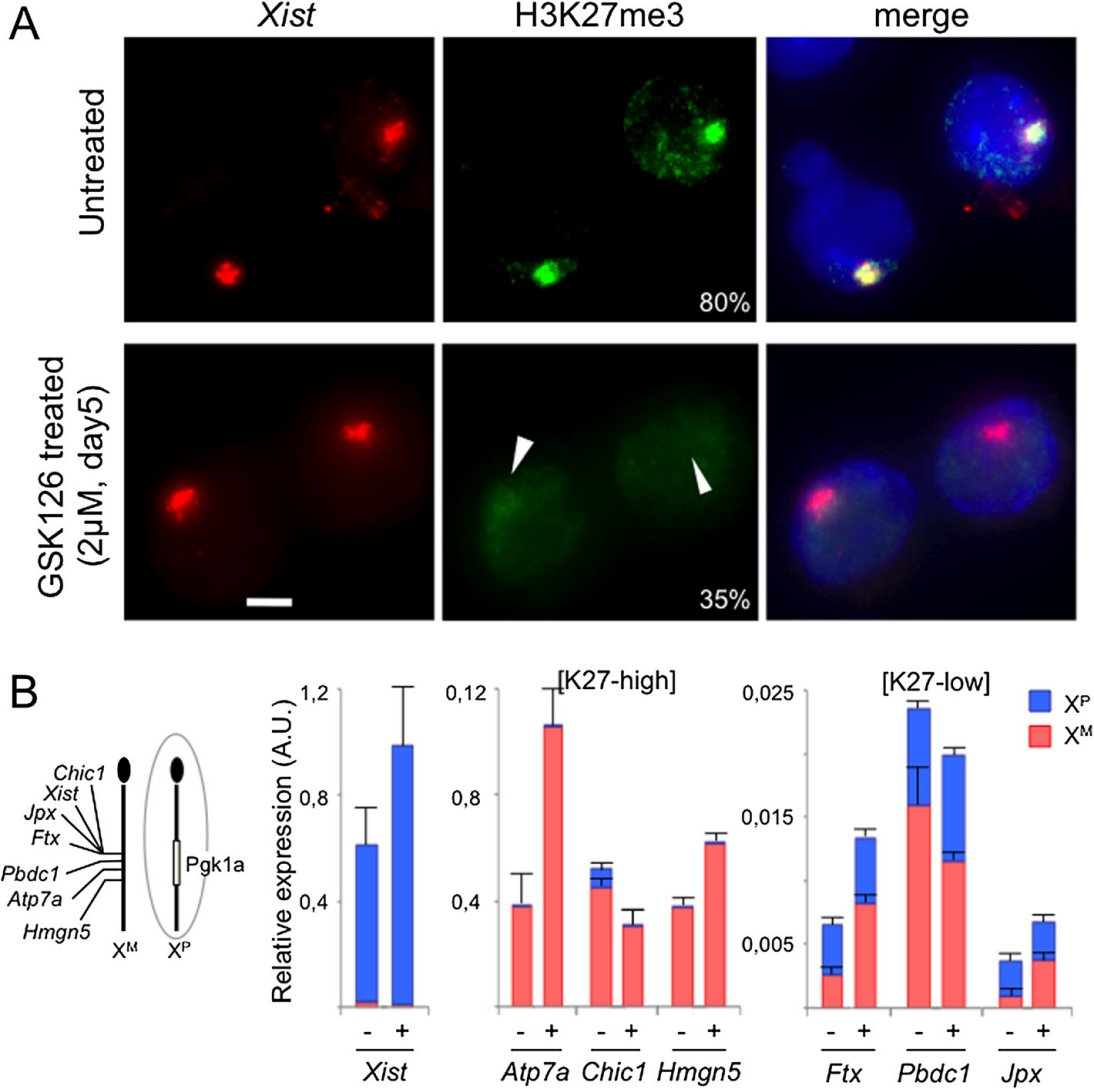
**X-linked gene expression in female XEN cells treated with the EZH2 inhibitor GSK126. (A)** Representative images produced by immunostaining followed by fluorescence *in situ* hybridisation on RNA for H3K27me3 (green) and *Xist* (red) on female extraendoderm stem (XEN) cells (GHP7/9 cell line) treated or not with 2 μM GSK126 for 5 days. Arrowheads indicate the nuclear position of the inactive X chromosome coated with *Xist* RNA. The percentage of visible H3K27me3 accumulation on the *Xist*-coated X chromosome in each condition is indicated. *n* > 50. Scale bar = 5 μm. **(B)** Cumulative histograms showing allelic gene expression levels of *Xist*, *Atp7a*, *Chic1*, *Hmgn5*, *Jpx*, *Ftx* and *Pbdc1* in female XEN cells (GHP7/9 cell line) treated (+) or not (−) with 2 μM GSK126 for 5 days. Mean values and standard deviations of two independent experiments are shown for each gene. Values have been standardised by the ubiquitously expressed *Rplp0* gene. On the left, the diagram shows the position of the genes analysed by reverse transcription followed by quantitative polymerase chain reaction along the X chromosome. No significant difference in the relative levels of paternal X (Xp) chromosome expression could be detected between treated and untreated cells (*P* > 0.05 by Χ^2^ test). AU, Arbitrary unit; [K27-high], Enriched in H3K27me3 in female compared to male XEN cells; [K27-low], Low levels of H3K27me3 in female and in male XEN cells; Xm, Maternal X chromosome.

In order to address whether the molecular mechanism controlling X-linked gene silencing and/or escape in XEN cells involves exclusively the specific distribution of H3K27me3 along the Xi, XEN cells were treated with GSK126, a potent and specific inhibitor of EZH2, the PRC2 catalytic subunit responsible for H3K27me3 apposition [32]. Importantly, the inhibitor had no visible impact on XEN cell morphology or growth rate and did not significantly change the expression of XEN-specific markers, indicating that GSK126 did not have important pleiotropic effects on XEN cell homeostasis (Additional file 7A and C). Western blot analysis of H3K27me3 on GSK126-treated compared to untreated XEN cells showed that the EZH2 inhibitor induced a progressive reduction in the global levels of H3K27me3, leading to barely detectable H3K27me3 levels after 5 days of treatment (Additional file 7B). Immunofluorescence analysis of H3K27me3, coupled with RNA-FISH for *Xist*, confirmed that most XEN cells (65%) treated with GSK126 lacked H3K27me3 accumulation on the Xi (*Xist*-coated X) after 5 days treatment (Figure 5A).

To assess the effect of GSK126-induced depletion of H3K27me3 on the expression of genes on the Xi, we analysed the expression of *Xist*, three genes subjected to I-XCI (*Atp7a*, *Chic1* and *Hmgn5*) and three genes escaping from I-XCI (*Ftx*, *Pbdc1* and *Jpx*) using allelic RT-qPCR on pools of XEN cells treated for 5 days with the drug. The expression levels of some of the X-linked genes, including *Xist*, were significantly increased after GSK126 treatment. Importantly, this increase affected exclusively alleles that were already active prior to any treatment—that is, the paternal *Xist* allele and maternal alleles of some other X-linked genes—suggesting that the inhibitor treatment leads to further derepression of active promoters (Figure 5B). In contrast, no significant change in expression levels of Xp alleles (except from *Xist*, as mentioned previously) was observed in GSK126-treated compared to untreated XEN cells, indicating that the marked reduction of H3K27me3 on the Xi of XEN cells did not significantly modify the efficiency of Xi gene silencing (Figure 5B). Similar results were obtained with two other H3K27me3-low genes (*Kdm6a* and *Nkap*) at the single-cell level using RNA-FISH following H3K27me3 immunostaining (Additional file 7D and E). Note that neither of the two analyses completely excludes the possibility that highly localised H3K27me3 may have remained associated with gene promoters, after GSK126 treatment, in some cells.

This analysis shows clearly that, although H3K27me3 enrichment allows the selective identification/segregation of genes subjected to I-XCI compared to genes escaping from I-XCI, this histone mark is probably not, by itself, determinant for X-linked gene expression control. This implies that other epigenetic marks or transcriptional mechanisms might be implicated in the regulation of Xi expression in XEN cells.

### Escape from imprinted X-inactivation is not modified upon XEN cell conversion into visceral endoderm

**Figure 6:**
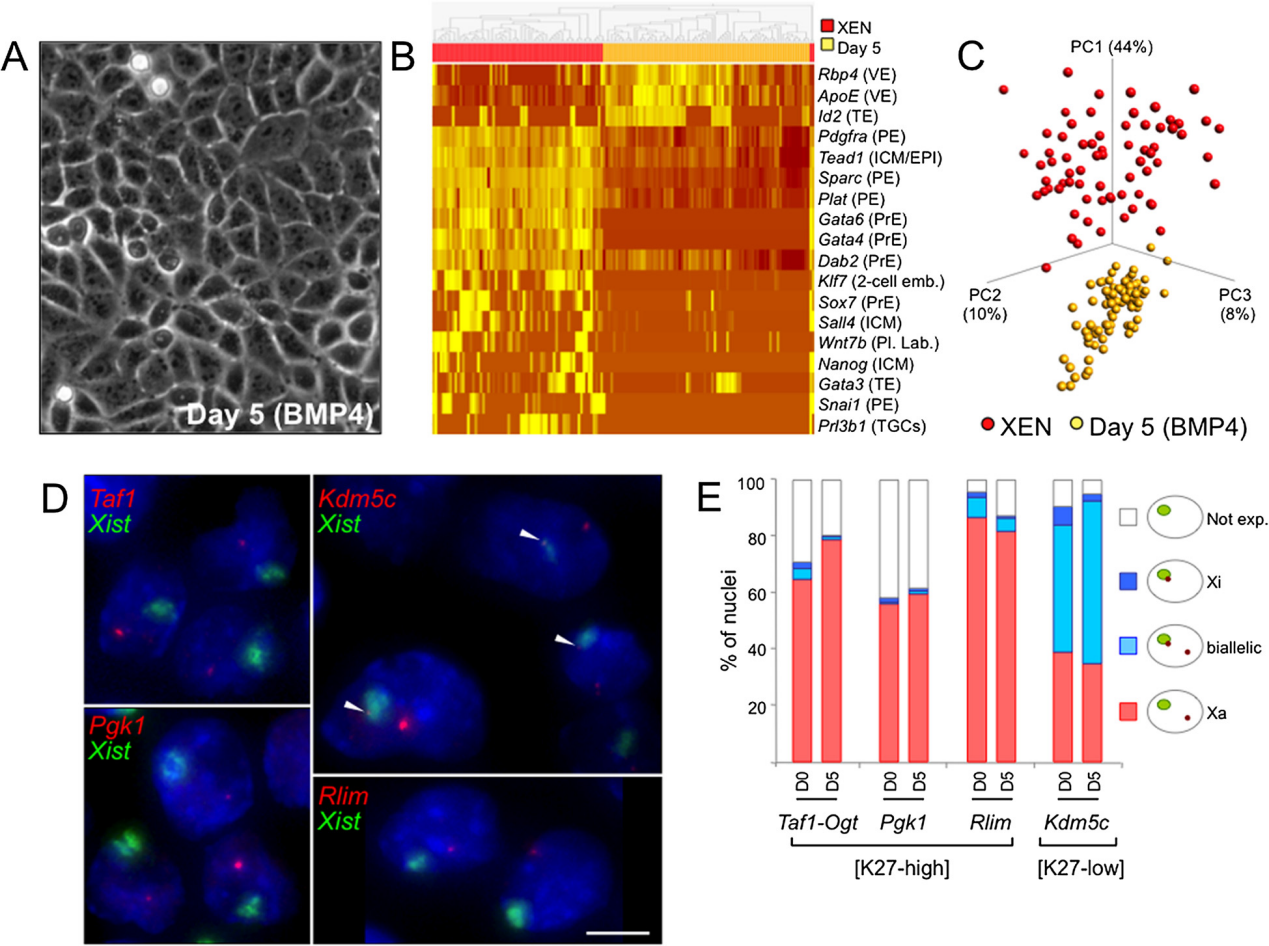
**X-linked gene expression upon conversion of XEN cells into visceral endoderm. (A)** Representative photographs illustrating the epithelium-like cell morphology observed after 5 days of bone morphogenetic protein 4 (BMP4) induction of extraendoderm stem (XEN) female cells (GHP7/9 cell line) are shown. **(B)** Heatmap of single-cell, steady-state RNA levels for the 18 most stringent discriminants of cells converted into visceral endoderm and undifferentiated XEN cells is shown (*P* < 10^−3^ by *F*-test). Hierarchical clustering has been applied. The complete data set is available in Additional file 2E. *n* = 72 female XEN cells (red) and *n* = 82 XEN cells treated with BMP4 (25 μM) for 5 days (yellow). The expected tissue specificity of each gene is indicated: EPI, Epiblast; ICM, Inner cell mass; PE, Parietal endoderm; Pl. Lab, Placental labyrinth; PrE, Primitive endoderm; TE, Trophectoderm; TGC, Trophoblast giant cell; VE, Visceral endoderm. The colour scale is the same as the one shown in Figure 1A. **(C)** Three-dimensional projections of principal components (PCs) 1, 2 and 3 of single-cell expression profiles of genes shown in **(B)**. **(D)** Two-colour fluorescence *in situ* hybridisation on RNA analysis for the indicated genes in XEN cells treated with BMP4 for 5 days. Arrowheads point to transcription signals associated with the *Xist*-coated inactive X chromosome. Scale bar = 5 μm. **(E)** Cumulative histogram showing the percentage of nuclei with the depicted expression pattern in XEN cells treated with BMP4 for 5 days (D5). Results obtained in untreated XEN cells (D0) are also shown to facilitate the comparison. No significant differences between X-linked gene expression profiles in BMP4 treated compared to untreated XEN cells could be detected (Χ^2^ test). *n* > 100. [K27-high], Enriched in H3K27me3 in female compared to male XEN cells; [K27-low], Low levels of H3K27me3 in female and in male XEN cells; Not exp., genes not significantly expressed; Xa, Active X; Xi, Inactive X.

Because observations of expression of genes from the Xp have previously been reported in the VE of EED mutants [24,33], we hypothesized that this extraembryonic tissue might be prone to labile Xp expression. Thus, we treated our female XEN cells with bone morphogenetic protein 4 (BMP4), a factor known to trigger XEN differentiation towards the VE fate [34,35], and then assessed the status of transcription at the Xi. After 5 days of induction, XEN cells formed a homogeneous, epithelium-like layer of cells characteristic of VE conversion (Figure 6A). Our expression analysis of lineage determinants at the single-cell level shows that the vast majority of cells had repressed PrE- and PE-specific markers and activated VE-specific genes, thus confirming the VE differentiation (Figures 6B and C and Additional file 2E). Transcription from the Xi, which we assessed by RNA-FISH, was not significantly induced upon VE conversion at the H3K27me3-high genes *Taf1*, *Rlim* and *Pgk1* or at the H3K27me3-low gene *Kdm5c* (Figures 6D and E). This observation indicates that, unlike what we saw during TS cell differentiation (Additional files 2E and 8), no change in Xi expression—at least for the genes we tested—is associated with XEN differentiation into VE.

## Discussion

Until now, it has been unclear whether differences in the silencing of genes on the Xi in the extraembryonic TE lineage, compared to tissues such as liver or embryonic kidney [31], are linked to the implementation of different mechanisms of X inactivation (that is, imprinted versus random) or whether they are associated with tissue specificity. In this study, we identified a large number of genes that escape from I-XCI solely in female XEN cells and not in female TS cells. Unlike the relaxation of I-XCI observed upon TS/TE cell differentiation (Additional file 8 and [16]), we observed that gene expression profiles from the inactive Xp in XEN cells appeared to be tightly maintained upon XEN conversion into VE cells *in vitro.* Hadjantonakis *et al*. made the same observation for yolk sac formation *in vivo*[19]. These observations strongly support the existence of distinct regulation of Xp silencing in each extraembryonic lineage of the blastocyst. According to our single-cell RT-qPCR analysis, the expression of I-XCI escapers does not correlate or anticorrelate significantly with the expression of any of the lineage biomarkers we tested. This is true in both XEN and TS cells, suggesting either that other, as yet unidentified regulators must be involved and/or that tissue specificity is established after higher-level regulation of these factors (Additional file 2B). For instance, such tissue specificity could be mediated by differences in the topological distribution of some of these factors along the inactive Xp in the PrE compared to the TE. Specific clustering of I-XCI escapers along the X chromosome was not observed in XEN cells, which is suggestive of gene-centred regulation (Additional file 2C). Gene-specific, *cis*-acting regions, either promoting escape from XCI or preventing the spreading of active chromatin into adjacent silent domain, have been identified in cells subject to random XCI, suggesting that similar *cis*-acting sequences may be recruited in extraembryonic tissues [36-38]. Adjacent ncRNAs and/or CTCF binding (CCCTC-binding factor (zinc finger protein)) may participate in insulating XCI escapers from globally inactive chromatin [39-44].

Another important observation concerns the variation in the degree of escape from gene to gene and, for certain genes, between XEN cell lines. Such variability has been reported, albeit to a lesser extent, in other tissues and organisms [31,45-48]. This characteristic may be symptomatic of an accrued lability in the silencing of discrete genomic locations and/or a manifestation of dynamic transitions between on/off states at paternal alleles, as observed previously in TS cells [18]. Ontology analyses of XEN-specific escapers did not reveal any striking overrepresentation of particular classes of biological function compared to that of genes stably subjected to I-XCI. Neither were we able to identify genes known to be involved specifically in yolk sac metabolism or development amongst the XEN-specific escapers. This could support the hypothesis that genes that are not crucial to XEN cell homeostasis are able to tolerate low levels of biallelic expression, whereas genes critical for XEN function might require tight control of their expression level. More detailed gene-by-gene functional studies are needed to definitively address this point and the role of such large-scale escape in PrE lineage development.

Our results show that the repressive histone mark H3K27me3 accumulates on the Xi in XEN cells in a manner similar to that observed in other cellular contexts, notably in its preferential targeting to the body of inactivated genes and in its exclusion from XCI escapers [17,29,31,49,50]. Strikingly, catalytic inactivation of EZH2 does not significantly affect the silencing of Xp genes in XEN cells, suggesting that H3K27me3 is not—at least not on its own—responsible for the maintenance of Xp gene silencing in XEN cells. Rather, the low levels of H3K27me3 at I-XCI escapers may be a by-product of the initial definition of loci escaping from XCI that is not critical in itself, but could reinforce the boundaries between active and silent chromatin along the inactive Xp. Because the disruption of PRC2 through the mutation of EED is associated with instability of I-XCI in extraembryonic derivatives [23,24], this result also implies that a function of the complex distinct from its H3K27me3 activity could be responsible for the maintenance of Xp-linked silencing. Other PRC2 members or cofactors, such as JARID2, which has recently been shown to bridge PRC2 to *Xist* RNAs, may be involved [51,52]. PRC1-mediated repressive marks, DNA methylation or macroH2A1 are other obvious candidates for serving this function [11,53]. Alternatively, active histone marks such as H3K4me2 or associated histone acetylation may positively target XEN escapers.

## Conclusions

Taken together, our results show that I-XCI in extraembryonic endoderm stem cells does not possess characteristics identical to X inactivation in other extraembryonic lineages or adult somatic tissues. Unlike the situation in TE cells, I-XCI in XEN cells is stably maintained in fully differentiated cells. We identified a large number of XEN-specific X inactivation escapers, which indicates that a mechanism dedicated to the definition of loci resistant to the global Xp silencing is at play in XEN cells. Importantly, this mechanism does not, at least not completely, rely on a XEN-specific distribution of H3K27me3. The next goal will be to unravel the nature of this mechanism and how it is recruited during initial cell commitment to the extraembryonic endoderm lineage.

## Methods

### Cell culture, drug treatment and *ex vivo* differentiation

Male GHP7/7 and female GHP7/9 and GHP7/3 XEN cell lines used in this study and have been described previously [26]. GHP7/9 and GHP7/3 cell lines carry an Xp from a 129.Pgk1a origin and an Xm from a 129/Sv origin, providing polymorphisms allowing for allelic distinction. XEN cells were grown on gelatin-coated culture dishes and routinely cultured at 37°C in 8% CO_2_ in Dulbecco’s modified Eagle’s medium (DMEM) (Life Technologies, Carlsbad, CA, USA) supplemented with 15% foetal bovine serum (Life Technologies) and 100 mM β-mercaptoethanol [26]. XEN cell conversion into visceral endoderm was induced upon addition of 25 ng/ml BMP4 (R&D Systems, Minneapolis, MN, USA) in serum containing DMEM [34]. XEN cell treatment with GSK126 was carried out as follows: three concentrations of inhibitor were tested during a 5-day treatment period (0.5 μM, 2 μM and 3 μM GSK126). Male F2 and female F3 TS cell lines and culture conditions have been described previously [12,18].

### ChIP-chip procedure and data analysis

ChIP assays were performed as previously described [54] using an H3K27me3 antibody (07–449) and an H3K4me2 antibody (07–030; both from EMD Millipore, Billerica, MA, USA) and analysed as described previously [18] (see also Additional file 9 for the detailed protocol). ChIP-chip data have been deposited under Gene Expression Omnibus accession number [GSE:50587].

Raw data were analysed using the Tobias Straub protocol (http://www.epigenesys.eu/images/stories/protocols/pdf/20111025114444_p43.pdf) with the Bioconductor R interface. This protocol includes quality assessment, data normalization and transformation. Raw H3K27me3 data in male and female livers were taken from the Gene Expression Omnibus database [GSE:20617] [31] and subjected to the same statistical treatment as the TS and XEN data sets. The gene expression data of TS and XEN cells were extracted from [GSE:15519] [29], and gene expression data of liver cells were extracted from [GSE:13583] [55].

### RNA-FISH and Immuno-RNA-FISH

RNA-FISH and immuno-RNA-FISH procedures were carried out as described at http://www.springerprotocols.com/Abstract/doi/10.1007/978-1-59745-406-3_18?verPrint=print[56] (H3K27me3 antibody, 07–449; EMD Millipore). For details of our experimental procedures, see Additional file 9. For probe localisation, see Additional file 2D. Z-stacks were captured (step = 0.2 μm) on a Zeiss Axioplan 2 microscope (Carl Zeiss Microscopy, Thornwood, NY, USA) equipped with a Hamamatsu ORCA ER charge-coupled device camera (Hamamatsu City, Japan) and controlled using Volocity Acquisition software (PerkinElmer, Waltham, MA, USA). Z-stacks deconvolution consisted in 50 iterations per each channel using Huygens Professional software (Scientific Volume Imaging, Hilversum, the Netherlands). Volume measurements were performed using Volocity Professional software, and intensity plot profiles were obtained on an image analysis platform in ImageJ software (National Institutes of Health, Bethesda, MD, USA).

### Single-cell gene expression analysis

Single-cell gene expression analysis was performed as described previously [18,57,58] and as recommended by Fluidigm (http://www.fluidigm.com/single-cell-expression.html; South San Francisco, CA, USA). Briefly, TS and XEN cells were sorted by fluorescence-activated cell sorting using the MoFlo system (Beckman Coulter, Brea, CA, USA), and individual cells were distributed into wells or 96-well plates containing 5 μl of CellsDirect resuspension buffer (Invitrogen, Carlsbad, CA, USA). The preamplification step consisted of 20 cycles using a mix of universal primer pairs to preamplify each gene simultaneously. Preamplification was followed by exonuclease I treatment (New England Biolabs, Ipswich, MA, USA), and allelic qPCR was performed on a BioMark thermal cycler (Fluidigm). Raw efficiencies of each PCR assay and allelic specificity were measured on control DNA within each experiment. Transcript levels were extrapolated using the raw PCR efficiencies, thus allowing the direct comparison of different genes. Controls for allelic specificities of PCR assays are available upon request. See Additional file 2A for primer sequences.

## Abbreviations

CHART-seq: Capture hybridisation analysis of RNA targets with deep sequencing; ChIP-chip: Chromatin immunoprecipitation followed by chip hybridisation; FRAP: Fluorescence recovery after photobleaching; ICM: Inner cell mass; I-XCI: Imprinted X chromosome inactivation; ncRNA: Noncoding RNA; PE: Parietal endoderm; PrE: Primitive endoderm; RAP: RNA antisense purification; RNA-FISH: Fluorescence *in situ* hybridisation on RNA; RT-qPCR: Reverse transcription followed by quantitative polymerase chain reaction; TE: Trophectoderm; TS cell: Trophoblast stem cell; VE: Visceral endoderm; Xa: Active X; XEN cell: Extraendoderm stem cell; Xi: Inactive X; Xm: Maternal X; Xp: Paternal X

## Competing interest

The authors declare that they have no competing interests.

## Authors’ contributions

SM carried out some of the ChIP-chip experiments, the ChIP data analysis, the FISH and immunofluorescence studies, the single-cell RT-qPCR studies, and the XEN differentiation experiments and inhibitor treatments, and also participated in manuscript writing. JLD established the protocol for the single-cell RT-qPCR analysis, designed some of the RT-qPCR primers and helped to draft the manuscript. AD performed the initial cell culture and characterisation of XEN cell lines before the ChIP procedure. PN established the protocol for ChIP on XEN cells and performed the initial ChIP-chip experiments. PA participated in the design and coordination of the study and helped to write the manuscript. CM coordinated the overall experiment design, participated in data interpretation, designed and tested the allelic PCR assays, participated in immuno-RNA-FISH studies and FISH data analysis and wrote the manuscript. All authors read and approved the final manuscript.

## Supplementary Material

Additional file 1 **Single-cell expression of lineage biomarkers in TS, XEN and ES female cells. (A)** At 3.5 to 4.5 days postcoitum, trophoblast stem (TS) cells and extraembryonic endoderm (XEN) stem cells can be derived from the polar trophectoderm and from the primitive endoderm, respectively. Representative photographs of TS (F2 male cell line) and XEN cells (GHP7/9 female cell line) illustrating the distinct morphologies of the two cell types. XEN cells are highly motile and do not require cell-cell contact to proliferate. As expected, they exhibit two distinct morphologies: (1) round-shaped refractile and (2) epithelium-like [26]. **(B)** and **(C)** Three-dimensional projections of principal components (PC) **(B)** and heatmap **(C)** of single-cell steady-state RNA levels for indicated lineage biomarkers (16 biomarkers analysed). Hierarchical clustering shows significant segregation between the three cell populations (*P* < 10^−5^ by *F*-test). ES cells were grown in 2i plus LIF medium [59]. Heatmap colour scale is the same as that shown in Figure 1A in the main text. *n* = 65 female TS cells, *n* = 72 female XEN cells and *n* = 37 female ES cells. Click here for file

Additional file 2 **(A) Primers used in single-cell gene expression analyses. (B)** Single-cell gene expression data in undifferentiated TS and XEN cells. **(C)** Expression of, and H3K27me3 percentage enrichment and H3K4me2 percentage enrichment of, X-linked genes in male and female TS and XEN cells. **(D)** Genomic regions covered by the FISH probes used in the study. **(E)** Single-cell gene expression data in TS cells differentiated for 3 or 6 days and in XEN cells treated with BMP4 for 5 days. Click here for file

Additional file 3 **H3K27me3 nuclear organisation on the inactive X chromosome in female TS cells. (A)** Representative image of immuno-RNA-FISH for H3K27me3 (green) and *Xist* (red) on female TS cells (F3 cell line). Quantification of fluorescence intensities for *Xist* and H3K27me3 across the inactive X domain show that the two domains do not strictly overlap. Maximal projections after deconvolution are shown. Scale bar = 5 μm. **(B)** Pie chart showing the percentage of nuclei exhibiting accumulation of *Xist* RNA only (red), coaccumulation of *Xist* RNA and H3K27me3 (yellow) or accumulation of H3K27me3 only (green) in female TS cells. **(C)** Boxplots showing the distribution of volumes occupied by *Xist* RNA and by H3K27me3 on the inactive X chromosome territory in female TS cells. The two distributions are significantly different (*P* < 0.05 by Kolmogorov–Smirnov test). *n* > 50. Click here for file

Additional file 4 **Distribution of H3K27me3 along the X-chromosomes in XEN and TS cells and in adult liver. (A)** to **(C)**. Scatterplots of H3K27me3 percentages along the body of expressed X-linked genes in female cells/tissues (*y*-axis) relative to male cells/tissues (*x*-axis). Each dot represents a single gene and its respective percentage of H3K27me3 in the corresponding cell line or tissue. *k*-means clustering was applied, which led to the identification of the same three classes of genes shown in Figure 2C in the main text: [0] = brown dots; [K27me3-high] = red dots and [K27me3-low] = green dots. Underneath the scatterplots, the pie charts show the percentage of expressed genes of each H3K27me3 class in each cell type. The number of expressed genes in each cell type is indicated (*n*). Liver ChIP-chip data were extracted from Gene Expression Omnibus ID [GSE:20617] [31] and subjected to the same statistical analysis as the ChIP-chip data obtained for TS and XEN cells. Note that only 376 X-linked genes have been analysed in the liver [31] compared to 642 X-linked genes in TS and XEN cell lines. We found a significantly higher percentage of expressed X-linked genes showing low levels of H3K27me3 in extraembryonic stem cells (either XEN or TS cells) compared to liver cells (*P* < 0.05 by Fisher’s exact test). In contrast, we observed no significant difference between the two XEN cell lines or between either XEN cell line and TS cells (*P* < 0.05 by Fisher’s exact test). Underneath the liver scatterplot, the Venn diagram shows the distribution of [K27-low] genes in TS cells, XEN cells and adult liver. This Venn diagram includes only X-linked genes that are expressed in all three cell types and that are common to the present study as well as to analyses of liver cells [31]. Expression data for TS and XEN cells were extracted from Gene Expression Omnibus ID [GSE:15519] [29]. XEN cell lines: male GHP7/7 vs. female GHP7/3; TS cell lines: male F2 vs. female F3. Click here for file

Additional file 5 **Distribution of H3K4me2 along the X chromosome in XEN cells. (A)** Boxplots showing the H3K4me2 distribution along expressed X-linked genes (Exp.) or along genes that are not significantly expressed (Not exp.) in male and female XEN cells (GHP7/7 and GHP7/9 cell lines). Similar results were obtained with female XEN cells (GHP7/3 cell line; not shown). Expression data were extracted from Gene Expression Omnibus ID [GSE:15519] [29]. *n* = 642 X-linked genes. *P < 0.05 by Kolmogorov–Smirnov test. **(B)** Representative examples of H3K4me2 distribution along [K27-high] and [K27-low] genes in male and female XEN cells. *mm9* UCSC screenshots. **(C)** Scatterplots of H3K4me2 percentages along the body of expressed X-linked genes in female (*y*-axis) relative to male (*x*-axis) XEN cells. Each dot represents a single gene and its respective percentage of H3K4me2 in the corresponding cell line. [K27-low] genes are shown in blue. The dotted line marks equal H3K4me2 percentages in male and female cells. Genes below the line are depleted in female compared to male cells, as expected for genes subject to XCI. In contrast, genes located around the line show similar levels of H3K4me2 in male and female cells, suggesting a biallelic enrichment in H3K4me2. [K27-low] genes are significantly enriched in H3K4me2 compared to [K27-high] genes in female XEN cells (*P* < 0.05 by Χ^2^ test). Click here for file

Additional file 6 **Single-cell RT-qPCR analysis on XEN cells. (A)** Representative heatmap of a single-cell RT-qPCR BioMark chip from Fluidigm. Each row represents a single XEN cell (72 in total), and each column a specific PCR assay. This Chip shows the quantification (cycle threshold (Ct) values) of two different reporter genes, twelve different allelic assays for X-linked genes (P, paternal X-specific; M, maternal X-specific amplifications) and fifteen different assays for lineage-specific markers. Negative controls include reactions in which the reverse transcriptase has been omitted (RT−), reactions in the absence of cell (TE control) and internal controls for allele specificity of each X-linked assay performed on genomic DNA (gDNA) of either paternal (Pgk1a) or maternal (129) origin. **(B)** Cumulative histograms showing allelic gene expression levels of *Xist*, *Atp7a*, *Jpx*, *Ftx* and *Pbdc1* on mixed populations of XEN cells (GHP7/9 cell line). Expression levels are standardised by the expression level of the reporter gene *Rplp0*. AU, Arbitrary unit. Note that these levels of expression are in agreement with single-cell expression of the same genes shown in Figure 3 in the main text. Click here for file

Additional file 7 **H3K27me3 abrogation upon treatment of female XEN cells with the EZH2 inhibitor GSK126. (A)** Representative images of female XEN (GHP7/9) cells treated or not with 2 μM GSK126 for 5 days. No morphological differences are apparent after 5 days of treatment with 2 μM GSK126. **(B)** Western blot analysis of H3K27me3 in XEN female cells (GHP7/9) treated with increasing concentrations of GSK126 for 1, 3 or 5 days. Total histone H3 detection is shown as a loading control. −, untreated; +, treated with GSK126. **(C)** RT-qPCR analysis of lineage markers in XEN cells (GHP7/9) treated with 2 μM GSK126 for 3 or 5 days. Mean ± standard deviation values of the relative expression levels of each indicated gene are shown. Values have been standardised by the ubiquitously expressed *Rplp0* gene. *n* = 2 independent experiments. ICM, Inner cell mass marker; PE, Parietal endoderm marker; PrE, Primitive endoderm marker; TE, Trophectoderm marker; VE, Visceral endoderm marker. No significant differences in the expression of most lineage markers were observed before and after treatment with GSK126, indicating that XEN cells retained their PrE identity. Only *Fst* was significantly downregulated after GSK126 treatment (*P* < 0.05 by Χ^2^ test). We do not have any explanation for this result. **(D)** Representative images of RNA-FISH following H3K27me3 immunostaining (green) analysis in XEN female cells (GHP7/9 cell line) treated with GSK126 (2 μM) for 5 days. The inactive X chromosome is detected by *Xist* RNA accumulation (red). Primary transcription at the indicated X-linked gene is codetected in yellow. Scale bar = 5 μm. **(E)** Cumulative histograms of the percentages of nuclei with the depicted expression pattern. Only nuclei showing a complete lack of H3K27me3 accumulation at the inactive X territory (*Xist*-coated X) are scored. X-linked genes are grouped according to H3K27me3 level. On the left of the histogram, the diagram shows the localisation of RNA-FISH probes along the X chromosome. No significant difference in the expression pattern could be detected between treated and untreated cells (*P* < 0.05 by Χ^2^ test). *n* > 50. Click here for file

Additional file 8 **X-linked gene expression in differentiating female TS cells. (A)** Representative photographs illustrating the different cell morphologies observed after 3 days (left panel) and after 6 days (right panel) of differentiation of female TS cells (F3 cell line). At day 6, TGCs (*) are clearly visible. **(B)** Heatmap of single-cell, steady-state RNA levels for the 20 most stringent discriminants of differentiated and undifferentiated TS cells (*P* < 10^−3^ by *F*-test). Hierarchical clustering has been applied. Complete data set is given in Additional file 2E. *n* = 65 female TS cells (green), *n* = 54 TS differentiated for 3 days (light blue) and *n* = 46 female TS cells differentiated for 6 days (dark blue). The expected tissue specificity of each gene is indicated. PE, Parietal endoderm; VE, Visceral endoderm; ICM, Inner cell mass; EPI, Epiblast; TE, Trophectoderm; Pl. Lab, Placental labyrinth; TGCs, Trophoblast giant cells; EPC, Ectoplacental cone. The colour scale is the same as that in Figure 1A in the main text. **(C)** Three-dimensional projections of principal components (PCs) of single-cell expression profiles of genes shown in **(B)**. **(D)** Two-colour RNA-FISH analysis of the indicated genes in TS cells differentiated for 3 days. The arrowhead points to a *Kdm5c* transcription signal on the *Xist*-coated inactive X chromosome that is not readily visible. Scale bar = 5 μm. **(E)** Three-colour RNA-FISH analysis of the indicated genes in TS cells differentiated for 5 days. Arrowheads point to *Rlim* and *Kdm5c* transcription signals on the *Xist*-coated inactive X chromosome that are especially difficult to see. Scale bar = 5 μm. **(F)** Cumulative histogram of the percentage of nuclei with the depicted expression pattern in TS cells differentiated for 3 days (D3) or for 5 days (D5). Results obtained in undifferentiated TS cells (D0) are also shown to facilitate the comparison. The asterisks mark significant differences between X-linked gene expression profiles in differentiated compared to undifferentiated TS cells (*P* < 0.05 by Χ^2^ test). *n* > 100. Click here for file

Additional file 9 Additional Methods. Click here for file
