# Supplementary figures and images for: Lineage-specific regulation of imprinted X inactivation in extraembryonic endoderm stem cells
Source: Epigenetics Chromatin. 2014 Jun 20;7:11. doi: 10.1186/1756-8935-7-11 (PMC4105886; doi:10.1186/1756-8935-7-11)

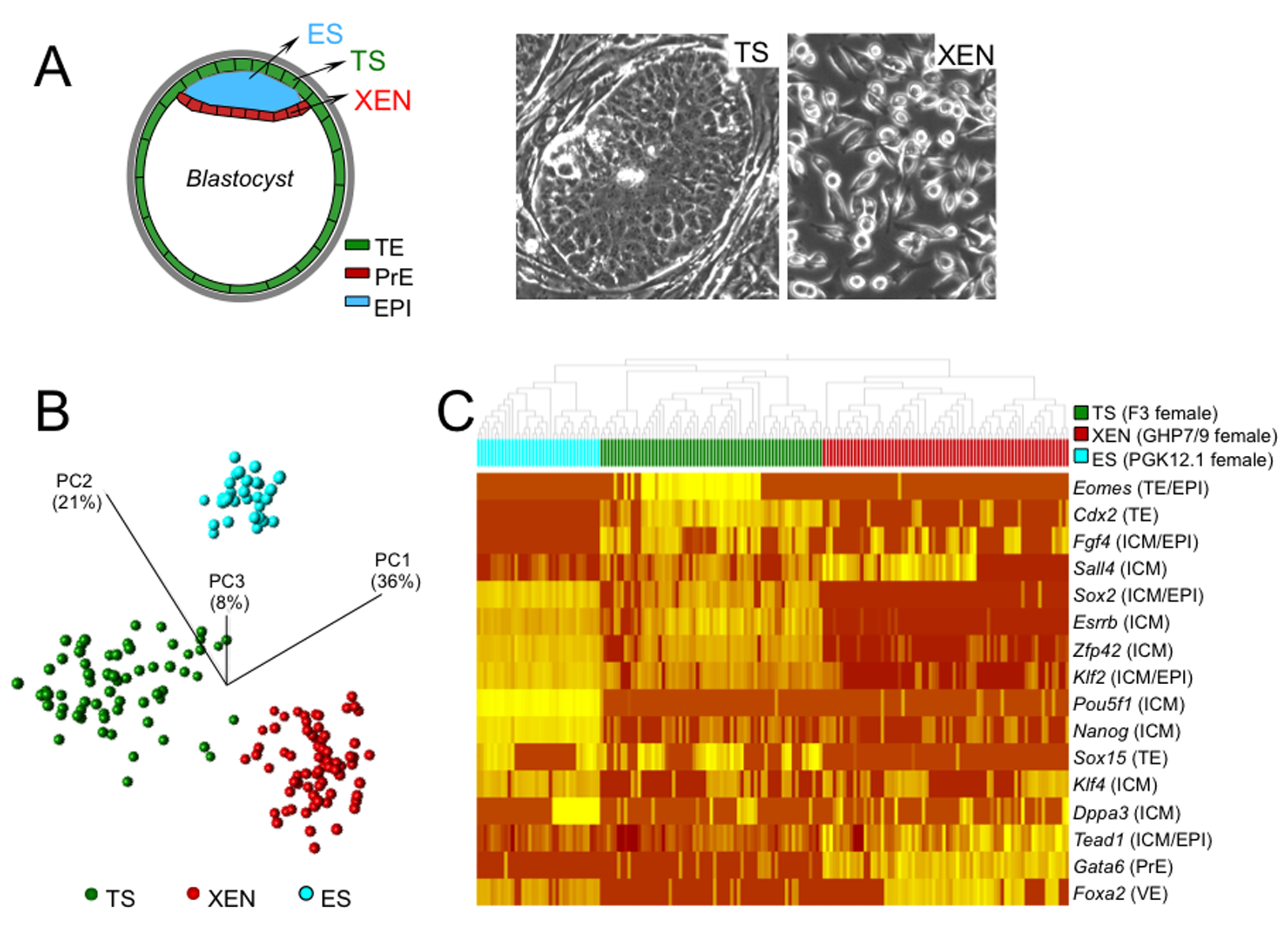

Supplement: Additional file 1 — Single-cell expression of lineage biomarkers in TS, XEN and ES female cells. (A) At 3.5 to 4.5 days postcoitum, trophoblast stem (TS) cells and extraembryonic endoderm (XEN) stem cells can be derived from the polar trophectoderm and from the primitive endoderm, respectively. Representative photographs of TS (F2 male cell line) and XEN cells (GHP7/9 female cell line) illustrating the distinct morphologies of the two cell types. XEN cells are highly motile and do not require cell-cell contact to proliferate. As expected, they exhibit two distinct morphologies: (1) round-shaped refractile and (2) epithelium-like [26]. (B) and (C) Three-dimensional projections of principal components (PC) (B) and heatmap (C) of single-cell steady-state RNA levels for indicated lineage biomarkers (16 biomarkers analysed). Hierarchical clustering shows significant segregation between the three cell populations (P < 10−5 by F-test). ES cells were grown in 2i plus LIF medium [59]. Heatmap colour scale is the same as that shown in Figure 1A in the main text. n = 65 female TS cells, n = 72 female XEN cells and n = 37 female ES cells. [file 1756-8935-7-11-S1.tiff]

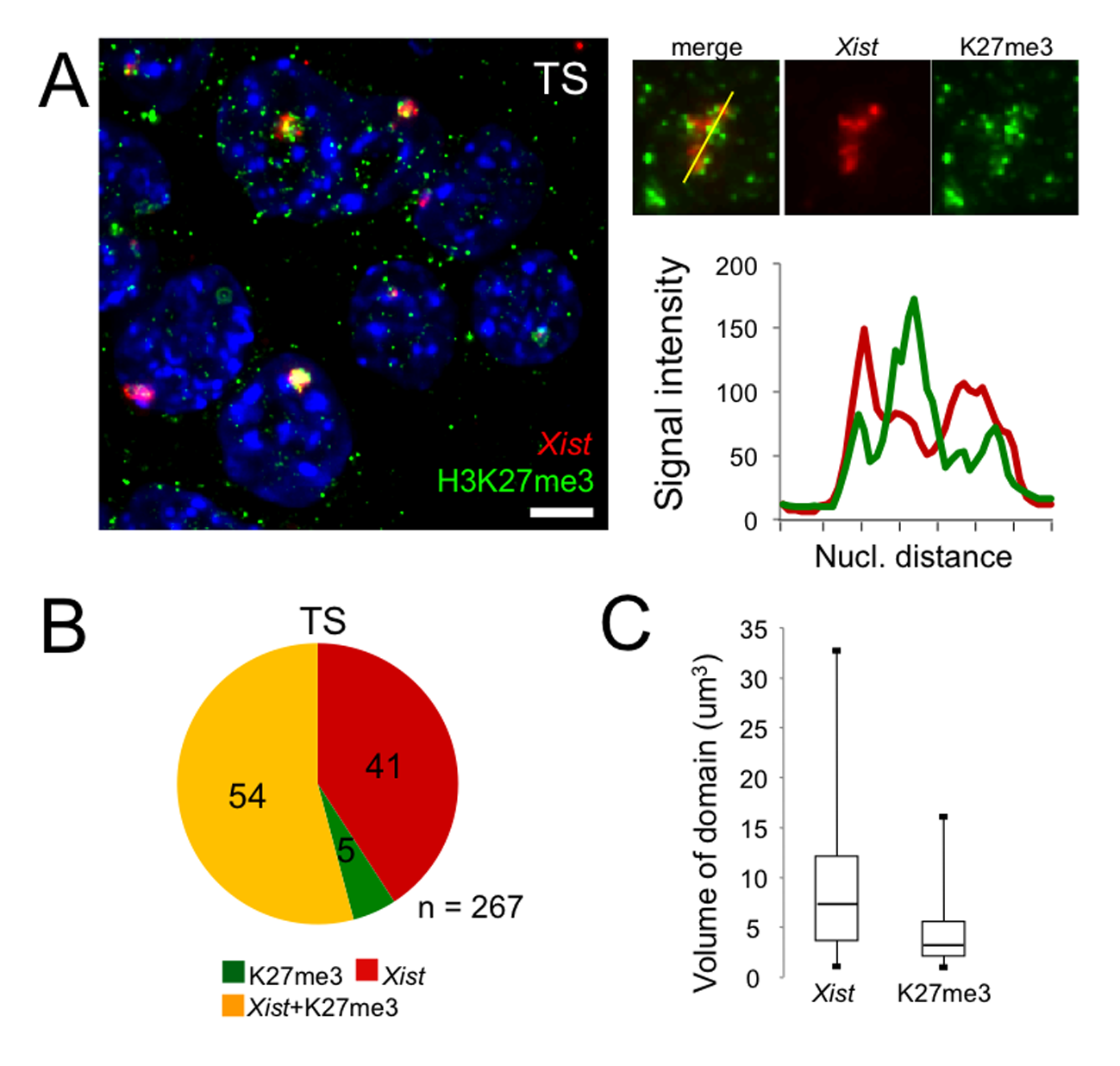

Supplement: Additional file 3 — H3K27me3 nuclear organisation on the inactive X chromosome in female TS cells. (A) Representative image of immuno-RNA-FISH for H3K27me3 (green) and Xist (red) on female TS cells (F3 cell line). Quantification of fluorescence intensities for Xist and H3K27me3 across the inactive X domain show that the two domains do not strictly overlap. Maximal projections after deconvolution are shown. Scale bar = 5 μm. (B) Pie chart showing the percentage of nuclei exhibiting accumulation of Xist RNA only (red), coaccumulation of Xist RNA and H3K27me3 (yellow) or accumulation of H3K27me3 only (green) in female TS cells. (C) Boxplots showing the distribution of volumes occupied by Xist RNA and by H3K27me3 on the inactive X chromosome territory in female TS cells. The two distributions are significantly different (P < 0.05 by Kolmogorov–Smirnov test). n > 50. [file 1756-8935-7-11-S3.tiff]

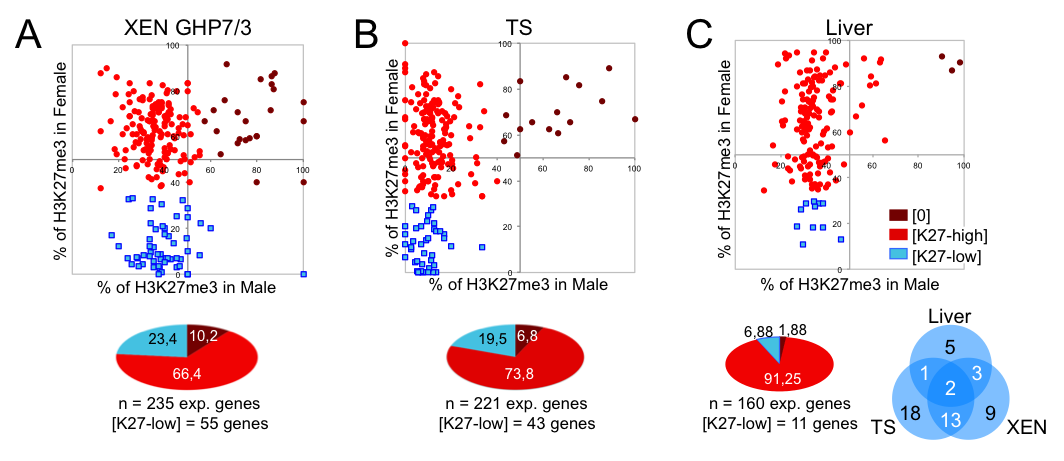

Supplement: Additional file 4 — Distribution of H3K27me3 along the X-chromosomes in XEN and TS cells and in adult liver. (A) to (C). Scatterplots of H3K27me3 percentages along the body of expressed X-linked genes in female cells/tissues (y-axis) relative to male cells/tissues (x-axis). Each dot represents a single gene and its respective percentage of H3K27me3 in the corresponding cell line or tissue. k-means clustering was applied, which led to the identification of the same three classes of genes shown in Figure 2C in the main text: [0] = brown dots; [K27me3-high] = red dots and [K27me3-low] = green dots. Underneath the scatterplots, the pie charts show the percentage of expressed genes of each H3K27me3 class in each cell type. The number of expressed genes in each cell type is indicated (n). Liver ChIP-chip data were extracted from Gene Expression Omnibus ID [GSE:20617] [31] and subjected to the same statistical analysis as the ChIP-chip data obtained for TS and XEN cells. Note that only 376 X-linked genes have been analysed in the liver [31] compared to 642 X-linked genes in TS and XEN cell lines. We found a significantly higher percentage of expressed X-linked genes showing low levels of H3K27me3 in extraembryonic stem cells (either XEN or TS cells) compared to liver cells (P < 0.05 by Fisher’s exact test). In contrast, we observed no significant difference between the two XEN cell lines or between either XEN cell line and TS cells (P < 0.05 by Fisher’s exact test). Underneath the liver scatterplot, the Venn diagram shows the distribution of [K27-low] genes in TS cells, XEN cells and adult liver. This Venn diagram includes only X-linked genes that are expressed in all three cell types and that are common to the present study as well as to analyses of liver cells [31]. Expression data for TS and XEN cells were extracted from Gene Expression Omnibus ID [GSE:15519] [29]. XEN cell lines: male GHP7/7 vs. female GHP7/3; TS cell lines: male F2 vs. female F3. [file 1756-8935-7-11-S4.tiff]

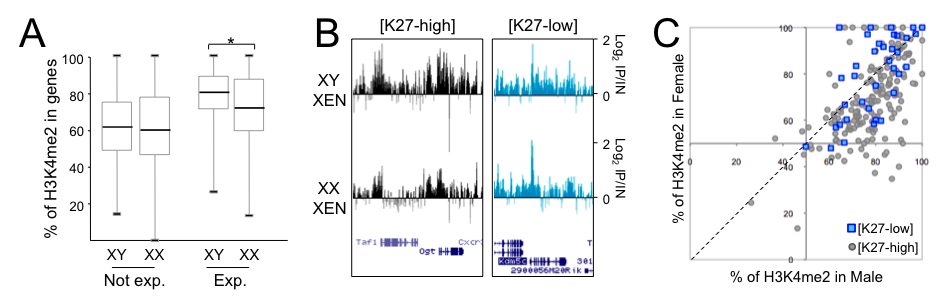

Supplement: Additional file 5 — Distribution of H3K4me2 along the X chromosome in XEN cells. (A) Boxplots showing the H3K4me2 distribution along expressed X-linked genes (Exp.) or along genes that are not significantly expressed (Not exp.) in male and female XEN cells (GHP7/7 and GHP7/9 cell lines). Similar results were obtained with female XEN cells (GHP7/3 cell line; not shown). Expression data were extracted from Gene Expression Omnibus ID [GSE:15519] [29]. n = 642 X-linked genes. *P < 0.05 by Kolmogorov–Smirnov test. (B) Representative examples of H3K4me2 distribution along [K27-high] and [K27-low] genes in male and female XEN cells. mm9 UCSC screenshots. (C) Scatterplots of H3K4me2 percentages along the body of expressed X-linked genes in female (y-axis) relative to male (x-axis) XEN cells. Each dot represents a single gene and its respective percentage of H3K4me2 in the corresponding cell line. [K27-low] genes are shown in blue. The dotted line marks equal H3K4me2 percentages in male and female cells. Genes below the line are depleted in female compared to male cells, as expected for genes subject to XCI. In contrast, genes located around the line show similar levels of H3K4me2 in male and female cells, suggesting a biallelic enrichment in H3K4me2. [K27-low] genes are significantly enriched in H3K4me2 compared to [K27-high] genes in female XEN cells (P < 0.05 by Χ2 test). [file 1756-8935-7-11-S5.tiff]

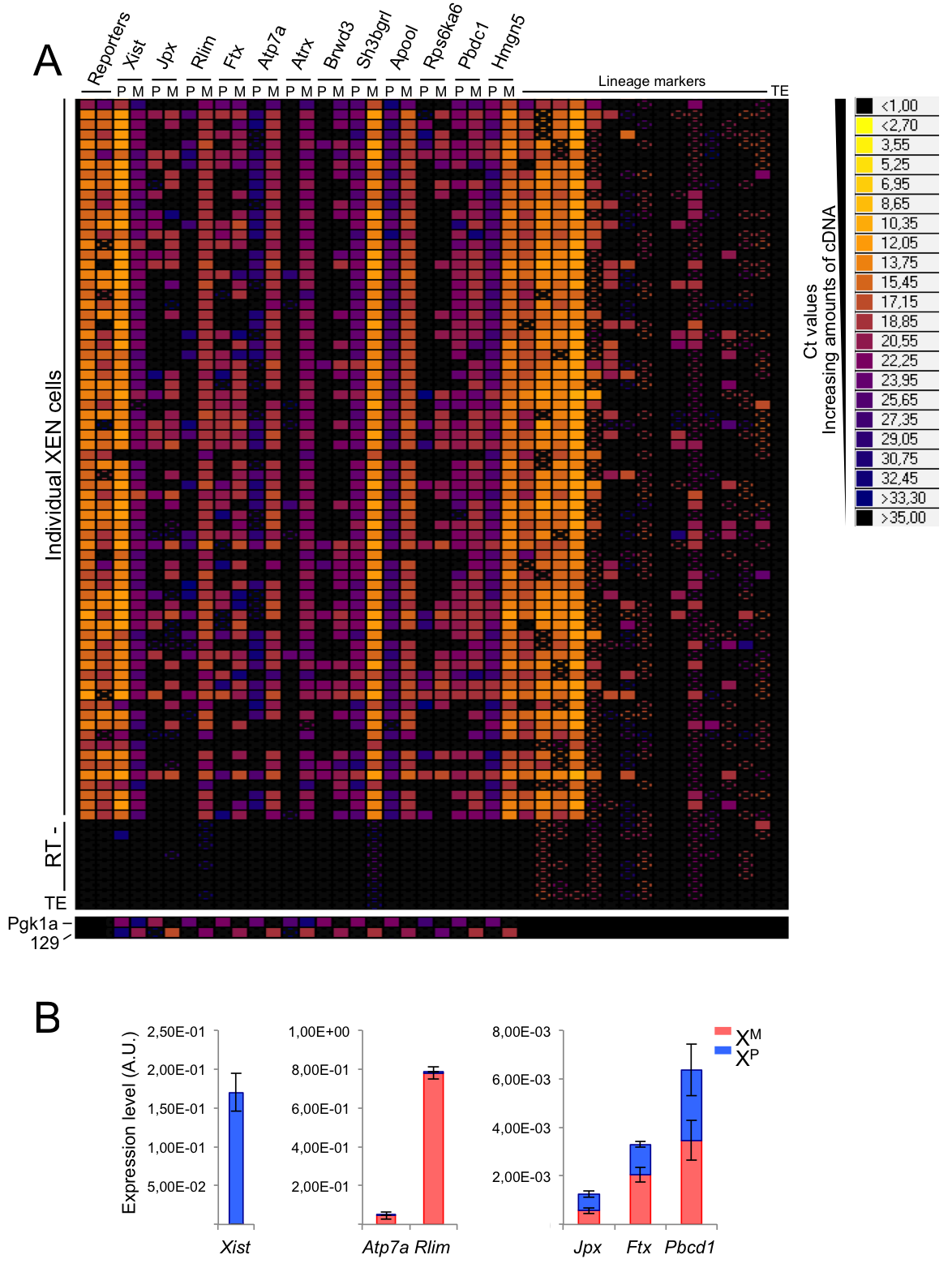

Supplement: Additional file 6 — Single-cell RT-qPCR analysis on XEN cells. (A) Representative heatmap of a single-cell RT-qPCR BioMark chip from Fluidigm. Each row represents a single XEN cell (72 in total), and each column a specific PCR assay. This Chip shows the quantification (cycle threshold (Ct) values) of two different reporter genes, twelve different allelic assays for X-linked genes (P, paternal X-specific; M, maternal X-specific amplifications) and fifteen different assays for lineage-specific markers. Negative controls include reactions in which the reverse transcriptase has been omitted (RT−), reactions in the absence of cell (TE control) and internal controls for allele specificity of each X-linked assay performed on genomic DNA (gDNA) of either paternal (Pgk1a) or maternal (129) origin. (B) Cumulative histograms showing allelic gene expression levels of Xist, Atp7a, Jpx, Ftx and Pbdc1 on mixed populations of XEN cells (GHP7/9 cell line). Expression levels are standardised by the expression level of the reporter gene Rplp0. AU, Arbitrary unit. Note that these levels of expression are in agreement with single-cell expression of the same genes shown in Figure 3 in the main text. [file 1756-8935-7-11-S6.tiff]

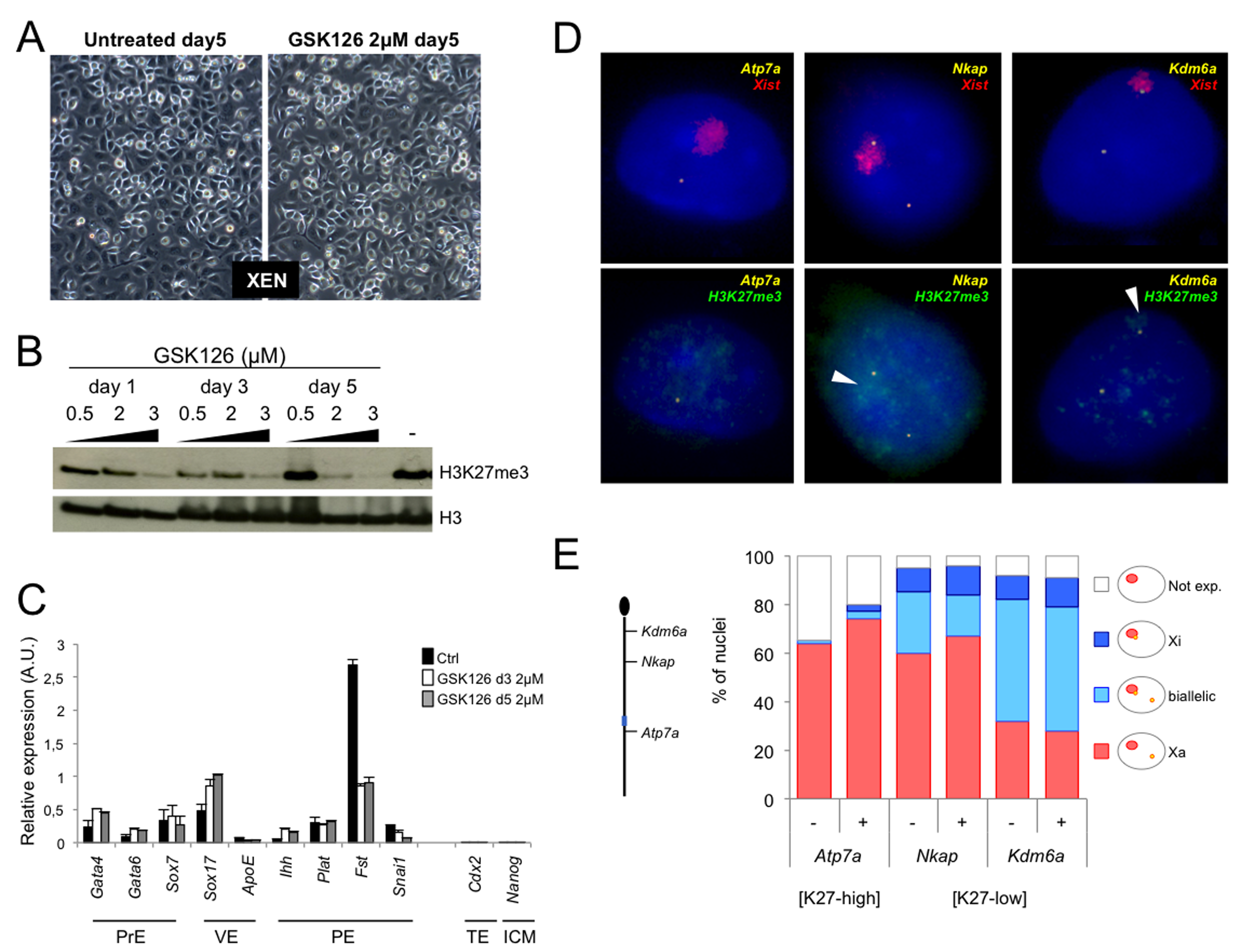

Supplement: Additional file 7 — H3K27me3 abrogation upon treatment of female XEN cells with the EZH2 inhibitor GSK126. (A) Representative images of female XEN (GHP7/9) cells treated or not with 2 μM GSK126 for 5 days. No morphological differences are apparent after 5 days of treatment with 2 μM GSK126. (B) Western blot analysis of H3K27me3 in XEN female cells (GHP7/9) treated with increasing concentrations of GSK126 for 1, 3 or 5 days. Total histone H3 detection is shown as a loading control. −, untreated; +, treated with GSK126. (C) RT-qPCR analysis of lineage markers in XEN cells (GHP7/9) treated with 2 μM GSK126 for 3 or 5 days. Mean ± standard deviation values of the relative expression levels of each indicated gene are shown. Values have been standardised by the ubiquitously expressed Rplp0 gene. n = 2 independent experiments. ICM, Inner cell mass marker; PE, Parietal endoderm marker; PrE, Primitive endoderm marker; TE, Trophectoderm marker; VE, Visceral endoderm marker. No significant differences in the expression of most lineage markers were observed before and after treatment with GSK126, indicating that XEN cells retained their PrE identity. Only Fst was significantly downregulated after GSK126 treatment (P < 0.05 by Χ2 test). We do not have any explanation for this result. (D) Representative images of RNA-FISH following H3K27me3 immunostaining (green) analysis in XEN female cells (GHP7/9 cell line) treated with GSK126 (2 μM) for 5 days. The inactive X chromosome is detected by Xist RNA accumulation (red). Primary transcription at the indicated X-linked gene is codetected in yellow. Scale bar = 5 μm. (E) Cumulative histograms of the percentages of nuclei with the depicted expression pattern. Only nuclei showing a complete lack of H3K27me3 accumulation at the inactive X territory (Xist-coated X) are scored. X-linked genes are grouped according to H3K27me3 level. On the left of the histogram, the diagram shows the localisation of RNA-FISH probes along the X chromosome. No significant differ [file 1756-8935-7-11-S7.tiff]

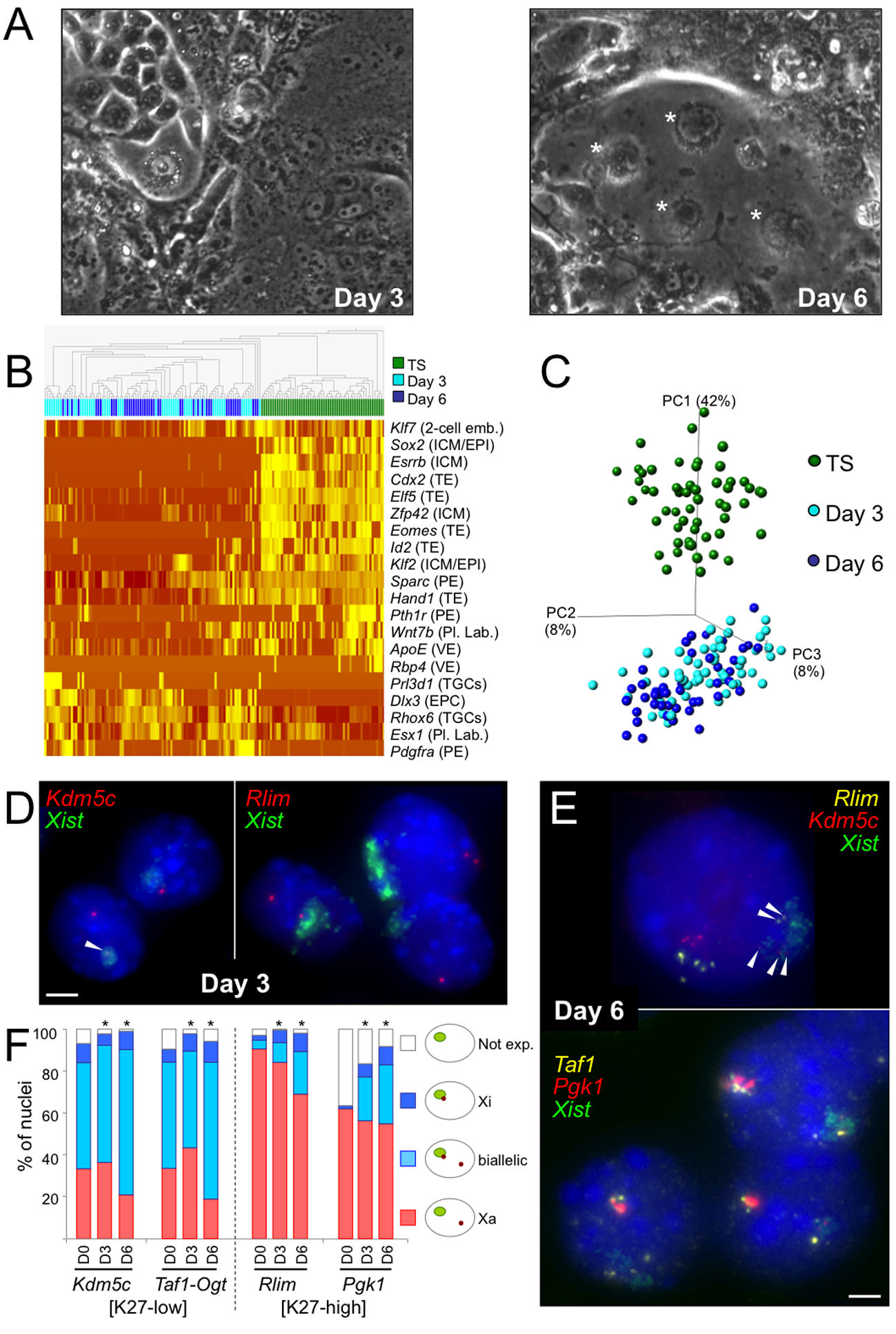

Supplement: Additional file 8 — X-linked gene expression in differentiating female TS cells. (A) Representative photographs illustrating the different cell morphologies observed after 3 days (left panel) and after 6 days (right panel) of differentiation of female TS cells (F3 cell line). At day 6, TGCs (*) are clearly visible. (B) Heatmap of single-cell, steady-state RNA levels for the 20 most stringent discriminants of differentiated and undifferentiated TS cells (P < 10−3 by F-test). Hierarchical clustering has been applied. Complete data set is given in Additional file 2E. n = 65 female TS cells (green), n = 54 TS differentiated for 3 days (light blue) and n = 46 female TS cells differentiated for 6 days (dark blue). The expected tissue specificity of each gene is indicated. PE, Parietal endoderm; VE, Visceral endoderm; ICM, Inner cell mass; EPI, Epiblast; TE, Trophectoderm; Pl. Lab, Placental labyrinth; TGCs, Trophoblast giant cells; EPC, Ectoplacental cone. The colour scale is the same as that in Figure 1A in the main text. (C) Three-dimensional projections of principal components (PCs) of single-cell expression profiles of genes shown in (B). (D) Two-colour RNA-FISH analysis of the indicated genes in TS cells differentiated for 3 days. The arrowhead points to a Kdm5c transcription signal on the Xist-coated inactive X chromosome that is not readily visible. Scale bar = 5 μm. (E) Three-colour RNA-FISH analysis of the indicated genes in TS cells differentiated for 5 days. Arrowheads point to Rlim and Kdm5c transcription signals on the Xist-coated inactive X chromosome that are especially difficult to see. Scale bar = 5 μm. (F) Cumulative histogram of the percentage of nuclei with the depicted expression pattern in TS cells differentiated for 3 days (D3) or for 5 days (D5). Results obtained in undifferentiated TS cells (D0) are also shown to facilitate the comparison. The asterisks mark significant differences between X-linked gene expression profiles in differentiated compared to undifferentiat [file 1756-8935-7-11-S8.tiff]
