# Supplementary material for: Lineage-specific regulation of imprinted X inactivation in extraembryonic endoderm stem cells
Source: Epigenetics Chromatin. 2014 Jun 20;7:11. doi: 10.1186/1756-8935-7-11 (PMC4105886; doi:10.1186/1756-8935-7-11)
Supplement: Additional file 9 — Additional Methods. [file 1756-8935-7-11-S9.docx]

ADDITIONAL METHODS

*TS and XEN cell culture and* ex vivo *differentiation*

# Undifferentiated TS cell lines were grown on plates containing mitomycin-treated Mouse Embryonic Fibroblasts (MEF) cells in presence of RPMI medium (Life technologies) complemented with 20% FBS (Life technologies), 1 mM sodium pyruvate, 2 mM L-glutamine, 50 mg/mL Penicillin/Streptomycin, 100 mM β-mercaptoethanol (all from Life technologies), 25 ng/mL hrFGF4 (Abcys P100-31) and 1 mg/mL heparin (Sigma-H3149) at 37^o^C in 5% CO_2_. For analysis, feeder cells were adsorbed on gelatin-coated culture dishes for 2h, at 37^o^C in 5% CO_2_. TS cells containing medium depleted from MEFs was then recovered. For single cell RT-qPCR experiments, FACS sorted and single cells were dispensed into wells of a 96-well plate containing 5 μL of CellsDirect extract buffer (Invitrogen).

# For ChIP analyses, TS cells were grown on culture dishes in 30% fresh TS medium and 70% of MEF conditioned TS medium.

# For FISH or immunostaining analyses, TS cells were plated on gelatin-coated SUPERFROST slides and grown for a day in 30% fresh TS medium and 70% of MEF conditioned TS medium.

# TS cell differentiation was induced through FGF4 and heparin removal [1].

*ChIP on Chip procedure*

ChIP on Chip procedure was as described in [1]. 10^7^ MEF-free TS cells were resuspended in 3 mL of DMEM with 10% FBS and crosslinked with 1% Formaldehyde solution (Sigma 37% stock solution) for 10 min at room temperature. Crosslinking was quenched with 125 mM Glycine (Sigma) for 5 min at room temperature and cells were washed twice in ice-cold PBS1X and cell lysis was performed in an ice-cold swelling buffer (5 mM PIPES, 85 mM KCl, 0,5% NP-40) supplemented with 1X Proteases inhibitors (Roche) for 20 min. Cells were spun down for 10 min at 400 g at 4°C and resuspended in ice-cold TSE150 buffer (150 mM NaCl, 20 mM Tris-HCl, 2 mM EDTA, 1% Triton X-100, 0,1% SDS) for sonication. The samples were sonicated using a Bioruptor (Diagenode) for 15 min with 30 sec ON/OFF cycles. Sonicated chromatin was checked on 1% agarose gel, divided into aliquots and stored at -80°C.

20 μg of sonicated chromatin per ChIP was incubated with 5 μg of H3K27me3 antibody (Upstate-Millipore, #07-449) or 5 μg of H3K4me2 antibody (Abcam, #ab7766) in 500 μl of TSE150 buffer at 4°C and rotated overnight. 50 μl of fresh 50% protein A beads solution were added and incubated for an additional 1.5 hour at 4°C. Beads were washed twice in TSE150, once in TSE500 (0,1% SDS, 1% Triton, 2 mM EDTA pH8, 20 mM Tris-HCl pH8, 500 mM NaCl), once in washing buffer (10 mM Tris-HCl, 250 mM LiCl, 0.5% NP-40, 0.5% Na-Deoxycholate, 1 mM EDTA) and twice in TE (10 mM Tris-HCl, 1 mM EDTA). Bound complexes were eluted from beads in 100 μl of Elution Buffer (50 mM Tris-HCl pH 8.0, 10 mM EDTA and 1% SDS) at 65°C for 15 min and washed in by TE 1% SDS. Crosslinking was reversed by overnight incubation at 65°C. Whole cell extract DNA reserved from the sonication step and corresponding to the Input (IN) material was also treated for crosslink reversal. Both IN and IP were Proteinase K treated and purified by standard Phenol:Chloroform extraction.

Whole genome amplification of both IP and IN was realised according to manufacturer recommendations (WGA-2 Sigma). The amplified DNA was labeled and hybridized by NimbleGen to a customised X-chromosome tiling array designed by Anton Wutz’s lab (Research Institute of Molecular Biology, Austria) and manufactured by NimbleGen System, Inc. according to NimbleGen standard protocols. A minimum of two replicates for each cell population isolated from independent cell cultures for each antibody were used for analyses.

*ChIP on Chip data analysis*

Reproducibility and quality control of the data were assessed using the “R” package combined with a protocol developed for ChIP-on-Chip data analysis of histone modifications (<http://www.epigenome-noe.net/WWW/researchtools/protocol.php?protid=43>). Briefly, the global hybridisation patterns across each array were controlled by MA-plotting of all the probe signals on the array and by calculating the Pearson correlation coefficients between pairs of replicates (all the coefficients were > 0.8). The two replicates showing the highest Pearson correlation coefficient were used for further analysis. For visualisation purposes Log_2_ (IP/IN) ratio datasets were converted into BED format and uploaded onto the UCSC Genome Browser.

A Lowess normalisation and “SAM” algorithms were applied to each dataset to determine statistically enriched probes [2]. X chromosome and chromosome 17 data were normalised separately. The percentage of statistically enriched probes across X-linked genes was then determined for each cell line and a cascade K-means clustering [3] was applied to expressed genes extracted from microarray expression data GSE15519 [4] using the cascadeKM function from the Vegan package (http://cran.r-project.org/web/packages/vegan/vegan.pdf). The number of groups for the partition was set from 2 to 10. 1000 iterations were tested. Three groups consistently emerged from this analysis, that were referred to as class (1), class (2) and class (3) genes. These classes gather genes enriched for H3K27me3 in males and females, genes enriched in H3K27me3 only in females “H3K27me3-high”, and genes lacking H3K27me3 in both males and females “H3K27me3-low” respectively.

*RNA-FISH and Immuno-RNA-FISH*

For RNA-FISH, MEFs-free TS and XEN cells (undifferentiated and differentiated) were resuspended at 3.10^5^ cells/spot in v/v DMEM/PBS and cytospun on slides for 4 min at 350 rpm. Slides were directly fixed with 3% PFA (pH 7.4) for 10 min at 4°C, permeabilised in PBS/0,5% Triton X-100 on ice for 5 min, and stored at 4°C in Ethanol 70%. Slides were then dehydrated with progressive 2 min Ethanol baths (70%, 90% and 100%) and then left to dry while precipitating the fluorescent probes. Labelled BAC or Fosmid probes (Nick translation kit, Abbott), were mixed with 3 μg of mouse Cot-1 DNA (Life technologies), 10 μg of salmon sperm DNA (Life technologies), 0.1 volume of 3M sodium acetate and precipitated in Ethanol for 15 min at -20°C. The pellet was washed twice with 70% Ethanol, resuspended in 7 μl of Formamide per spot (Sigma) and denatured for 10 min at 75°C. 7 μl of Hybridisation buffer was added to denatured probes, and the mixture was loaded on the dried slides and hybridized at 37°C overnight. Slides were then washed twice in 50% Formamide/2XSSC (pH7.4) at 39^o^C for 5 min, once in 2XSSC at 39°C for 5 min and mounted in DAPI-containing Vectashield (Vector Laboratories).

For Immuno RNA-FISH cells were directly grown on gelatin-coated slides (Superfrost) overnight. Slides were then fixed for 15 min at room temperature with 3% PFA (pH 7.4), washed twice in ice-cold PBS, and permeabilised for 12 min in PBS/0,5% Triton X-100 at room temperature. Cells were then incubated with 1/200 of the H3K27me3 primary antibody (Upstate/Millipore) and detected with an AlexaFluor 488 anti-rabbit (1/200, Molecular probes/Life technologies) secondary antibody. Slides were post-fixed with 3% PFA (pH 7.4) for 10 min at 4°C and hybridised as for regular RNA-FISH.

For immuno RNA-FISH, z-stacks were captured (step = 0,2 μm) on a Zeiss Axioplan2 microscope equipped with a Hamamatsu orca-ER CCD camera and controlled by the Perkin Elmer acquisition software, Volocity. Z-stacks deconvolution consisted in 50 iterations per each channel using the Huygens-Pro software (Scientific Volume Imaging). Volume measurements were performed on Volocity (Perkin Elmer) and intensity plot profiles obtained on image analysis platform ImageJ.

SUPPLEMENTAL METHODS REFERENCES

1. Dubois A, Deuve JL, Navarro P, Merzouk S, Pichard S, et al. (2014) Spontaneous reactivation of clusters of X-linked genes is associated with the plasticity of X-inactivation in mouse trophoblast stem cells. Stem Cells 32: 377-390.

2. Tusher VG, Tibshirani R, Chu G (2001) Significance analysis of microarrays applied to the ionizing radiation response. Proc Natl Acad Sci U S A 98: 5116-5121.

3. Calinski T, Harabasz J (1974) A dendrite method for cluster analysis. Commun Stat 3: 1-27.

4. Rugg-Gunn PJ, Cox BJ, Ralston A, Rossant J (2010) Distinct histone modifications in stem cell lines and tissue lineages from the early mouse embryo. Proc Natl Acad Sci U S A 107: 10783-10790.
